# Frege on intuition and objecthood in projective geometry

**DOI:** 10.1007/s11229-021-03080-0

**Published:** 2021-03-09

**Authors:** Günther Eder

**Affiliations:** Department of Philosophy, University of Vienna, Universitätsstrasse 7, 1010 Vienna, Austria

**Keywords:** Frege, Nineteenth century geometry, Projective geometry, Intuition

## Abstract

In recent years, several scholars have been investigating Frege’s mathematical background, especially in geometry, in order to put his general views on mathematics and logic into proper perspective. In this article I want to continue this line of research and study Frege’s views on geometry in their own right by focussing on his views on a field which occupied center stage in nineteenth century geometry, namely, projective geometry.

## Introduction

To most philosophers, Gottlob Frege is best known as one of the founding fathers of modern logic and for his contributions to the philosophy of logic, language and mathematics. But in the first instance, Frege was a professional mathematician whose main area of specialization was in geometry. One of the central developments in nineteenth century geometry was the rise of projective geometry. So it is interesting to see what Frege has to say on this crucial subject for at least three reasons. First, in light of the importance of the subject and the significance of Frege as a historical figure, it is intrinsically interesting to see what Frege thinks about this topic. Secondly, by looking at Frege’s works in geometry, we can learn something about the way in which geometry was practised in the nineteenth century. Finally, investigating Frege’s views on specific aspects of geometry is important in order to form an accurate picture of his overall views on mathematics and its philosophy. A lot of excellent work has been done in this area in recent years. Mark Wilson, Jamie Tappenden, Paolo Mancosu, Jeremy Shipley, Patricia Blanchette, and Matthias Schirn, to name a few, have all made important contributions to a re-evaluation of Frege’s views in light of his mathematical background.^1^ The aim of this paper is to deepen these investigations by bringing together existing research, expanding on some of the issues raised by these authors in connection with projective geometry, and by adding a couple of new ones that have been neglected so far. In this way, we want to form a comprehensive picture of Frege’s views on a topic that was considered to be crucial by nineteenth century geometers.

The plan for this article is as follows. The following section will provide a rough outline of the main threads in nineteenth century projective geometry. Section 3 will give an overview of the different stages in Frege’s thinking about geometry and set the stage for the subsequent discussion of specific issues related to projective geometry in Frege’s early works in Sects. 4–8. Sections 9–12 will be be concerned with the question how Frege’s views further developed in the *Grundlagen* and later on. In the final section, I conclude with a couple of general comments and some remarks on further topics to be investigated.

## Nineteenth century projective geometry

The development of mathematics in the nineteenth century is marked by a number of innovations and fundamental shifts. In geometry, a variety of new topics had been introduced and new geometries were emerging at a rapid rate. Geometers started to seriously investigate non-Euclidean geometries, geometries of more than three dimensions were being considered, imaginary forms had found their way into geometry, and topology started to become an independent discipline. Attempts to remove the shortcomings in Euclid’s *Elements* also prompted the development of modern axiom systems by the end of the century. In this article, however, I want to focus on one development in particular, namely, the rise of projective geometry, which is linked in various ways to almost all developments in geometry during the nineteenth century. The following is intended to give an informal outline for orientation. We will look at specific details once we get to Frege.^2^

**Fig. 1:**
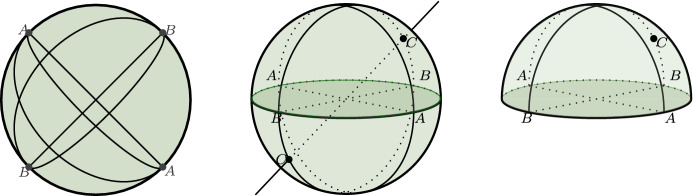
Intuitive picture of the real projective plane as a disk (left). The interior of the disk represents the Euclidean plane and the boundary circle the line at infinity. Points at infinity are represented by pairs of antipodal points on the boundary circle and lines that meet in antipodal points represent parallels. In the sphere-model (center), projective points are represented by pairs of antipodal points on the sphere and projective lines by great circles. In the semi-sphere model (right), projective points are represented by points on the sphere and pairs of antipodal points on the boundary circle. By orthogonal projection of the semi-sphere onto the plane of the boundary circle we get a precise version of the disk-model on the left

The origins of projective geometry lie in the renaissance study of central projections in connection with the study of perspective drawing, although central concepts of projective geometry can be found already in ancient geometry.^3^ Roughly, projective geometry studies the properties of figures that remain invariant under central projection and can be described as the result of two modifications of Euclidean geometry. First, since metrical properties like lengths, areas, or angles are not in general preserved by central projection, all of these properties are ruled out. Second, since parallel lines (i.e., lines that don’t meet) are not in general mapped to parallel lines, the notion of parallelism is ruled out as well. Instead, in projective geometry parallel lines are said to ‘meet’ at some ‘point at infinity’. So just as two points always determine a straight line, in projective geometry, two straight lines always determine a unique point, their common intersection point, although in some cases this will be a point at infinity. All points at infinity are then assumed to lie on a ‘line at infinity’, which somehow ‘surrounds’ the ordinary Euclidean plane (see Fig. 1, left).

Although people had been using projective methods and elements at infinity occasionally before, it was only at the beginning of the nineteenth century that projective geometry started to thrive as an independent discipline in the hands of geometers like Gaspard Monge, Jean Victor Poncelet, and Joseph Diez Gergonne. With his *Traité des propriétés projectives des figures* (Poncelet 1822), Poncelet gave projective geometry a general direction by showing that there is a part of geometry that does not rely on metrical considerations, but which is nonetheless rich and interesting. Poncelet was also one of the first to make systematic use of elements at infinity. Moreover, he also made a serious attempt to justify their introduction.^4^ Both elements at infinity and imaginary elements (i.e., geometrical objects that would be described in analytic geometry by means of complex coordinates) were recognized by Poncelet to be powerful extensions of traditional geometrical ontology. Inspired by Poncelet’s success, geometers after him gradually extended the realm of projective methods and started to revise definitions of concepts that relied on metrical notions whenever possible in favour of ones that did not rely on metrical notions.^5^ This trend towards purification found its ultimate expression in Karl Georg Christian Von Staudt’s *Geometrie der Lage* from 1847, whose stated aim was to “to establish the geometry of position as an independent science that does not need to rely on measurement” (Von Staudt 1847, V).

Projective geometry as it was conceived by Poncelet and many of his followers was considered to be an alternative mainly to *analytic* geometry, which had dominated geometry since Descartes. The heavy use of algebraic reasoning and mechanical calculation that is characteristic of analytic geometry was by many considered to be foreign to geometry proper. However, not everyone was following Poncelet in avoiding analytic methods. Indeed, some of the most important innovations in projective geometry came from the analytic tradition, after people like Möbius and Plücker introduced various systems of *homogeneous coordinates*.^6^ The introduction of homogeneous coordinates was a crucial innovation that furthered projective geometry in several ways. First and foremost, it made projective geometry amenable to rigorous, algebraic investigations. Just like in analytic Euclidean geometry, we can now translate problems of projective geometry into the language of algebra, do the calculations, and then translate the result back into the language of geometry without having to engage in unwelcome speculations about the nature of ‘points at infinity’. Points at infinity are represented by the same kind of objects as ordinary points, namely, ratios of numbers.

But there are several other advantages to the analytic point of view. For one, whereas Poncelet had to invoke his controversial ‘principle of continuity’ to account for imaginary forms, analytic geometry gets them for free. We merely have to interpret the numerical variables to range over complex numbers instead of real numbers. Another development that resulted from work in analytic projective geometry was Plücker’s idea to think of the coefficients in equations of geometrical objects as their coordinates, which also led to the idea of arbitrary ‘space elements’.^7^ The analytic approach to projective geometry also paved the way for various other developments, including Arthur Cayley’s discovery that, in a sense, metrical geometry is *contained* in projective geometry, which led to his notorious claim that “metrical geometry is thus a part of descriptive [i.e., projective, G.E.] geometry, and descriptive geometry is *all* geometry and reciprocally” (Cayley 1859, p. 90). Cayley’s results were subsequently extended by Felix Klein to cover non-Euclidean geometries as well, and also set into the broader context of his famous ‘Erlangen program’, in which various geometries are classified according to their associated transformation groups.^8^

The ‘projective point of view’ may have been merely a convenient way to organize Euclidean geometry for some geometers, but the real projective plane was a definite object of study that was also investigated topologically towards the end of the nineteenth century. The construction of mathematically precise models or representations of the projective plane was instrumental for this. We have already mentioned analytic representations in terms of homogeneous coordinates, which were a standard item in the tool kit of geometers by the middle of the nineteenth century. ‘Intuitive’ representations can be derived from homogeneous coordinates by interpreting point-triples as straight lines through the origin in three-dimensional space, line-triples as planes through the origin, and by stipulating that a projective point is incident with a line just in case the line corresponding to the point is contained in the plane corresponding to the line.^9^ From this ‘homogeneous model’ we get a bunch of related models by means of various projections, which were also investigated topologically during the second half of the nineteenth century (see Fig. 1, center and right).

Projective geometry reached its peak at the turn of the twentieth century, when geometers were beginning to investigate various axiom systems for projective geometry. The axiomatic treatment of projective geometry started with Moritz Pasch’s famous *Vorlesungen über neuere Geometrie* in 1882, and was refined by several Italian geometers in the 1890s, including Peano and other members of his group, and American ‘postulate theorists’ such as Veblen and Young. On the axiomatic approach, projective geometry is developed purely deductively based on a small number of basic concepts and postulates or axioms.^10^ Veblen and Young already follow the modern pattern in which geometrical axioms are understood as schemas that are satisfied or ‘realized’ by various models and where systems of homogeneous coordinates are conceived as standard models of the axioms of projective geometry. After this period, interest in projective geometry began to decline and projective geometry gradually lost its status as a foundation for all of geometry.

Again, this is just a very rough overview. For now, what is important is that projective geometry occupied a central place in nineteenth century geometry. As Felix Klein notes right at the beginning of his famous inaugural lecture from 1872: “Among the achievements of the past fifty years in geometry, the development of *projective geometry* occupies the first place.” (Klein 1872, p. 460) Frege was, among other things, a nineteenth century geometer. So what did Frege have to say on this crucial subject?

## Three periods in Frege’s thinking about geometry

Frege never made his views on projective geometry explicit. So in order to answer this question, we have to reconstruct his views from scattered remarks throughout his writings. But before we enter the details, let me make a couple of general remarks for orientation.

Generally speaking, one can divide Frege’s thinking related to geometry roughly into three periods according to their characteristic themes. The first may be labelled the *mathematical period*, the second the *philosophical period*, and the third the *foundational period*. The mathematical period stretches from the beginnings of Frege’s professional career until sometime between the publication of the *Begriffsschrift* in 1879 and the *Grundlagen der Arithmetik* in 1884, and it is dominated by Frege’s purely mathematical work in geometry. Frege, remember, was a trained mathematician and was just becoming a ‘Privatdozent’ (the rough equivalent of an assistant professor) for mathematics in Jena in 1874. As such, he lectured on both synthetic and analytic geometry, both in their traditional, Euclidean form and according to the ‘newer’, i.e., projective, method. Almost all of his published writings in this early period are related to geometry.^11^ Unfortunately, we don’t know much about the content of his lectures. So all we have are Frege’s doctoral and habilitation dissertations and a couple of articles, and it is these writings that we will focus on. As we will see, even though these writings contain little by way of explicit philosophical discussion, they are nonetheless rich enough to draw some interesting conclusions about Frege’s conception of geometry, particularly projective geometry.

The second period in Frege’s thinking about geometry starts sometime after the appearance of the *Begriffsschrift* and is centered around the *Grundlagen*. Generally speaking, this period is marked by two important developments. First, there is a growing interest in questions related to logic and its foundations, and, as a result of this, the creation of the Begriffsschrift as a formal system of logic. Secondly, and relatedly, we can also observe an increased interest in philosophical questions compared to the earlier, mathematical period. With respect to geometry in particular, this is seen most pointedly in the *Grundlagen*, where Frege draws on several specifically Kantian themes.

The last period is dominated by general methodological questions related to the foundations of geometry and mathematics more broadly. This period was triggered by the publication of Hilbert’s celebrated *Grundlagen der Geometrie* (Hilbert 1899), which led to the notorious Frege–Hilbert controversy, a debate that affected Frege’s thinking almost until the end of his life. In his correspondence with Hilbert, and subsequently in a series of articles titled “Über die Grundlagen der Geometrie” from 1903 to 1906, Frege criticizes Hilbert on various accounts, including his conceptions of axioms and definitions and his methodology of reinterpreting axioms in order to establish consistency and independence results. With the exception of a last-ditch effort towards the end of his life where Frege tries to ground arithmetic in geometry (Frege 1979, p. 278), his remarks on geometry typically revolve around the topics that were at issue in his controversy with Hilbert.

Although I will say a few things related to the foundational period, the bulk of what follows will be concerned with the first and the second period. This is because, for one, a lot has been said on the Frege-Hilbert controversy already.^12^ But, more importantly, since the last period revolves around general methodological questions in mathematics, with a few exceptions, it isn’t really helpful in understanding Frege’s views on geometry and its philosophy. With that said, let’s dive right in and see what early Frege has to say on elements at infinity.

## Elements at infinity

As we saw in Sect. 2, one of the central ideas of nineteenth century projective geometry was the introduction of elements at infinity: infinitely many points at infinity and a single line at infinity in the case of planar projective geometry, and infinitely many lines at infinity and a single plane at infinity in the case of solid projective geometry. Frege discusses elements at infinity as early as 1873 in his doctoral dissertation, where he compares elements at infinity with imaginary forms in geometry (more on imaginary elements and Frege’s dissertation shortly). The passage is worth quoting in full, since it already contains various themes that will be important later on. After noting that one might well question the sense of imaginary forms because they seem to “contradict all our intuitions”, Frege writes:

By way of comparison let us take forms at infinity, which do not occur in the space of intuition either. Taken literally, a ‘point at infinity’ is even a contradiction in terms; for the point itself would be the end point of a distance which had no end. The expression is therefore an improper [uneigentlich] one, and it designates the fact that parallel lines behave projectively like straight lines passing through the same point. ‘Point at infinity’ is therefore only another expression for what is common to all parallels, which is what we commonly call ‘direction’. As a straight line is determined by two points, it is also given by a point and a direction. This is only an instance of the general law that, whenever we are dealing with projective relationships, a direction can represent [vertreten] a point. By designating the direction as a point at infinity, we forestall a difficulty which would otherwise arise because of the need to distinguish a frequently unsurveyable set of cases according to whether two or more of the straight lines in the set were parallel or not. But once the principle of the equivalence of direction and point is established, all these cases are disposed of at one blow. (Frege 1984, p. 1)

There are several things in this passage that are worth emphasizing. First, Frege claims that elements at infinity do not belong to the “space of intuition”. Second, according to Frege, the expression “point at infinity” is an “improper” one and what is designated by this expression is more properly called a “direction”. And, third, designating a direction as a “point at infinity” is nevertheless useful because it helps to avoid various case distinctions and thereby furthers unification. Let’s start with the last point.

As we have hinted at in Sect. 2, the language of elements at infinity provides a powerful tool that furthers unification by eliminating exceptions. To illustrate, consider the following proposition:

Desargues’ theorem: If the lines joining corresponding vertices of two triangles meet in a point, then the intersection points of corresponding sides of the triangles lie on a line.

**Fig. 2:**
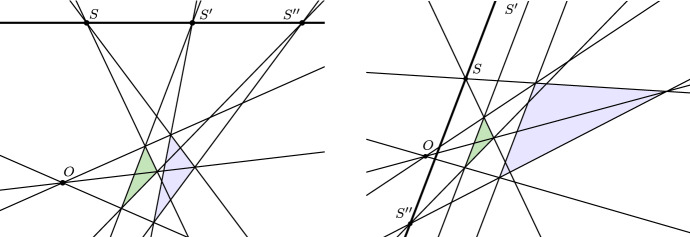
A Desargues configuration where corresponding sides of the triangles meet in ‘proper’ points (left), and one where corresponding sides are parallel (right)

Note that from a Euclidean point of view, this proposition really is not a ‘theorem’, because it depends on the additional assumption that the intersection points of corresponding sides of the two triangles *exist*, as is the case in the configuration on the left in Fig. 2. But what if this is not the case and certain pairs of corresponding sides are parallel as in the configuration on the right of Fig. 2? Interestingly, we can still prove a related theorem about configurations like these. Suppose, as stated in the antecedent of our original theorem, that the lines joining corresponding vertices of two triangles meet in a point. Furthermore, suppose that two corresponding sides of the triangles meet in a point *S* and that two further sides are parallel. Then, according to the new theorem, the intersection point $S''$ of the third pair of corresponding sides must lie on the line determined by *S* and the direction of the two parallel sides. Now, it may initially seem that this theorem is not really related to Desargues’ theorem as stated earlier. But if we adopt the language of elements at infinity and think of parallel lines as meeting at a ‘point at infinity’, then our original formulation of Desargues’ theorem covers the theorem about the configuration with parallel sides as well. It is just that the intersection point $S'$ is now a ‘point at infinity’ or a ‘direction’.^13^

This example nicely illustrates the kind of advantage that is gained by “designating the direction as a point at infinity” that Frege emphasizes in the last two sentences of the quoted passage. Elements-at-infinity-talk has a considerable instrumental value in organizing geometry, a fact that was stressed by most nineteenth century geometers. [See, e.g., the discussions in (Von Staudt 1847, pp. 23–25) or (Reye 1866, p. 15).] Indeed, throughout his doctoral dissertation (to which we turn shortly), Frege himself makes frequent use of this kind of talk. But by acknowledging the instrumental value of this way of speaking, Frege is not yet committed to any substantial views about the proper status of elements at infinity. This brings us to the other two points mentioned above. According to Frege, elements-at-infinity-talk, however useful, is also misleading, which is why he notes that the expression “point at infinity” is an “improper” one. It is not entirely clear if Frege uses the term “improper” merely to point to an unclarity in the *expression* “point at infinity”, or if he wants to suggest that the objects that are designated by this expression are “improper” themselves, either because they are non-intuitive or because they are not genuine objects in the first place. Either way, referring to elements at infinity as “improper” objects is by no means original to Frege and was common at the time.^14^ So while the term “improper” may suggest that Frege thinks that points at infinity have an ontological status that is somehow inferior to that of “proper” points, at this point, it is not clear just how much of this is merely loose talk on Frege’s part. Similarly, the term “direction”, which is proposed by Frege as a substitute for “point at infinity”, is not unprecedented either. In particular, Von Staudt had already used the term “direction” decades before Frege, noting that in many cases a direction can “represent” (“vertreten”) a point, and that referring to a direction as an infinitely distant point is “not inexpedient” because it highlights the commonalities between directions and ordinary points (Von Staudt 1847, pp. 23–24). In fact, Frege’s remarks on elements at infinity in his dissertation are so close to Von Staudt’s, both in spirit and wording, that it is plausible to assume that Frege specifically had Von Staudt’s book in mind when he was writing the introduction.^15^

In any case, while Frege tells us in his doctoral dissertation that points at infinity “do not occur in the space of intuition” and that they are more properly called “directions”, he does not tell us what these entities actually are (if they are genuine objects at all), why exactly he takes them to be non-intuitive, and what that even means. These questions are, at least in part, taken up again in 1884 in his *Foundations of Arithmetic*. But before we get to that, we will have a closer look at Frege’s doctoral dissertation from 1873.

## Frege’s doctoral dissertation

As indicated earlier, the inclusion of imaginary elements was an important development in nineteenth century geometry, and much of nineteenth century projective geometry can only be understood properly if we take geometers to be concerned with the complex projective plane rather than the real projective plane. With a few exceptions in the synthetic camp, imaginary elements were accepted by most geometers by the second half of the nineteenth century. So what did Frege think about imaginary forms in geometry?

Our main source on Frege’s views about imaginary forms is his doctoral dissertation from 1873, which also gives us insights into Frege’s views on several other themes.^16^ As the title suggests, the main aim of the dissertation is to provide a “geometrical representation of imaginary forms”. Before he explains what this is and how it is achieved, Frege discusses some general issues. He first mentions two ways to conceive of imaginary elements. The first is to introduce imaginary points by means of complex coordinates, and the second is to define imaginary points “in purely geometrical terms” (Frege 1984, p. 2). After providing a few hints related to the geometrical approach, Frege then focusses on the coordinate-approach. He notes that, with a few important exceptions, many of the laws that hold for the real numbers are still valid for the complex numbers. Hence, “the large extent to which imaginary forms conform to the same laws as real ones justifies the introduction of imaginary forms into geometry” (ibid.). Frege then says that imaginary forms are “improper elements” just like points at infinity, and that “there now arises the need, not only for treating these improper elements in the same way as the proper ones, but also for having them before our eyes” (ibid.). This is supposed to be achieved by providing a representation of imaginary forms in Euclidean space, which is “a kind of correlation in virtue of which every real or imaginary element of the plane has a real, intuitive element corresponding to it” (ibid.). The bulk of Frege’s dissertation is supposed to fill in the details. In what follows I will provide a brief exposition of some of his key ideas.

Frege’s general approach is as one would expect from his introductory discussion. He starts off by introducing imaginary points “in the way they are defined in algebraic analysis” (ibid., 3) and takes an imaginary point *P* to be given by rectangular coordinates (*x*, *y*), where $x = \xi + i \xi '$ and $y = \eta + i \eta '$ are complex numbers. In order to represent *P* in Euclidean space, Frege introduces two distinguished planes, the “plane of the real” and the “plane of the imaginary”. The two planes, which we designate by $\mathcal {R}$ and $\mathcal {I}$ in what follows, are assumed to be parallel to each other and each is assumed to be equipped with a rectangular coordinate system. The pair $(\xi , \eta )$ consisting of the real parts of *x* and *y* can then be identified with a point on $\mathcal {R}$, and the pair $(\xi ', \eta ')$ consisting of the imaginary parts with a point on $\mathcal {I}$. Thus, we can take the imaginary point *P* to be represented by a pair consisting of the points $(\xi , \eta )$ and $(\xi ', \eta ')$ or, as Frege prefers, by the line joining them.

**Fig. 3:**
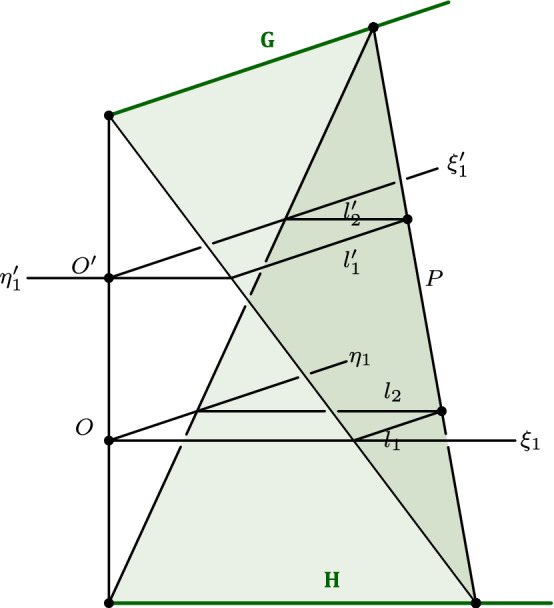
Frege’s representation of the imaginary straight line by guide lines $\mathbf{G}$ and $\mathbf{H}$. A point *P* on an imaginary line is represented by a line that joins a point on $\mathbf{G}$ with a point on $\mathbf{H}$ (or the corresponding pair of points in $\mathcal {I}$ and $\mathcal {R}$ that determine this line). (The diagram is an adaptation from Frege’s first diagram in his dissertation.)

Frege then goes on to consider more complicated imaginary forms. To get a sense of how this works, let’s look at the basic case of the imaginary straight line in more detail. Frege starts by considering the equation of the imaginary straight line

$$\begin{aligned} ux + vy + 1 = 0 \end{aligned}$$

where the coefficients *u* and *v* are assumed to be complex numbers. Applying some straightforward algebraic manipulations, it can be shown that this equation can be split up into two conditions of the form

$$\begin{aligned} \xi ' = A + B \xi + C \eta \quad \eta ' = D + E \xi + F \eta \end{aligned}$$

where the Latin uppercase letters represent real numbers that meet certain conditions that are determined by the complex coefficients *u*, *v* in the original equation of the straight line. If we conceive of these expressions as functions of $\xi$ and $\eta$, then via these “mapping functions” points in $\mathcal {R}$ are mapped to points in $\mathcal {I}$. Frege then introduces new coordinates $\xi _1, \eta _1$ and $\xi _1', \eta _1'$ for $\mathcal {R}$ and $\mathcal {I}$ respectively by applying a certain coordinate transformation, which leads to simpler mapping functions of the form

$$\begin{aligned} \xi _1' = A_1 + C_1 \eta _1 \quad \eta _1' = D_1 + E_1 \xi _1 \end{aligned}$$

where the Latin uppercase letters again represent real numbers that meet certain conditions. It is easy to see that these new mapping functions have the special property that the image of each line that is parallel to the $\xi _1$-axis will be mapped to a line that is parallel to the $\eta _1'$-axis, and that, similarly, each line that is parallel to the $\eta _1$-axis will be mapped to a line that is parallel to the $\xi _1'$-axis. Hence, by rotating the plane $\mathcal {I}$ by 90 degrees with regard to $\mathcal {R}$, Frege is able to arrange the setup so that parallels to the $\xi _1$-axis and parallels to the $\eta _1$-axis become parallel to their respective images under the new mapping functions (see Fig. 3).

As we saw, each imaginary point *P* can be identified with a pair of points $(\xi _1, \eta _1)$ and $(\xi _1', \eta _1')$, where the latter is the result of applying the new mapping functions to the point $(\xi _1, \eta _1)$. Each such pair of points determines two pairs of lines. The first pair consists of a line $l_1$ parallel to the first axis in $\mathcal {R}$ and its image $l_1'$ in $\mathcal {I}$, which, by the foregoing, is parallel to $l_1$. The second pair consists of a line $l_2$ parallel to the other axis in $\mathcal {R}$ and its image $l_2'$ in $\mathcal {I}$, which is parallel to $l_2$. It follows from the properties of the new mapping functions that all planes that are determined by a pair $(l_1, l_1')$ meet in a certain line **G** and that all planes that are determined by a pair $(l_2, l_2')$ meet in a certain line **H** (see Fig. 3). The lines **G** and **H** have the special properties that (i) they are perpendicular to each other, that (ii) they are both parallel to $\mathcal {R}$ and $\mathcal {I}$ and that (iii) the distance of **G** from $\mathcal {I}$ is the same as the distance of **H** from $\mathcal {R}$ (but in the opposite direction). If we trace back Frege’s definitions, we can see that the lines **G** and **H** (Frege calls them “guide lines”) are uniquely determined by our original equation of the imaginary straight line. Moreover, since the two guide lines determine all imaginary points on an imaginary straight line, Frege stipulates that “we regard them as a representation of the latter” (ibid., 7). So there we have it. In Frege’s construction, imaginary straight lines are represented by certain pairs of Euclidean straight lines. Conversely, as Frege points out, *every* pair of straight lines with the properties (i)–(iii) represents an imaginary line.

In the remaining parts of the dissertation, Frege discusses further topics, including the notion of the *distance* between two imaginary points and of the *angle* between two imaginary lines.^17^ He also generalizes his approach to provide representations of curves other than straight lines (ibid., 22 ff.). In particular, he discusses several issues relating to the representation of the *imaginary circle* (ibid., 30 ff.), including the circular points at infinity and the important case where a real line intersects a real circle in two imaginary points, noting that these are examples of “how our method can be used to represent non-intuitive relationships” (ibid., 34). For now, this should be sufficient to get a rough idea of Frege’s key ideas.

Now, to repeat the obvious, Frege’s principal approach to the representation of imaginary forms in his dissertation is based on purely *analytic* considerations. In the main part of his dissertation, imaginary points are explicitly introduced in terms of coordinates because this would allow us “to describe them in their entirety in the most general way” (ibid., 3). Various imaginary forms (straight lines, circles, etc.) are then introduced via their characteristic equations and, based on Frege’s initial framework, representations in Euclidean space are derived using algebraic manipulations, coordinate transformations, and the like. It is only once representations have been derived in this way that geometrical constructions in Euclidean space take over.

On the other hand, Frege does mention in the introductory section that imaginary points can also be defined “in purely geometrical terms”. He explicitly points out that this can be achieved “by combination of a circle with a straight line or by involution on the straight line” (ibid., 2). Here, Frege is apparently referring to Von Staudt’s famous definition of imaginary points from the sequel to his *Geometrie der Lage*, the *Beiträge zur Geometrie der Lage* from 1856, where Von Staudt presents an ingenious geometrical construction, where imaginary elements are defined in terms of real elements.^18^ Importantly though, Von Staudt and Frege are pursuing different projects. Whereas Von Staudt is concerned with some kind of *reduction* or *definition* of imaginary points in terms of purely geometrical constructions in order to avoid reference to complex coordinates, Frege in his dissertation is concerned with a visualization or “intuitive representation” of imaginary elements, where the latter are *defined* in terms of complex coordinates.^19^

Frege’s remarks concerning imaginary forms are admittedly vague enough to allow for different interpretations. For example, his remark that defining imaginary points in terms of coordinates “allows us to describe them in their entirety in the most general way” may well be read as an implicit acknowledgement that complex coordinates are merely a means to *describe* an underlying structure which itself is independent of these coordinates, much as the Euclidean plane is a structure that can be *described* in terms of real coordinates, but which is nonetheless independent of any particular coordinate system. So, on such a view, the ‘coordinate definition’ of imaginary points would be merely an analytic ‘representation’. Of course, then the question once again arises: What *are* imaginary points? Given his expressed view that imaginary forms are similar to elements at infinity in that both are “improper” elements, one may surmise that he might favour a similar explication as well. But to my knowledge, Frege nowhere indicates an unambiguous answer to this question. Even in the context of his detailed discussion of how points at infinity are to be treated in his *Grundlagen*, Frege does not mention imaginary elements.^20^

## Geometrical representation

We have seen that in his dissertation Frege maintains that both points at infinity and imaginary elements are “improper” elements that do not belong to the “space of intuition”, that is, Euclidean space. But that does not mean that we cannot *represent* these elements in various ways, including ones in which they are represented by intuitive objects. We have seen in some detail how Frege aims to achieve this for imaginary forms. We now want to look at a couple of questions that are raised by this and other examples of geometrical representation.

Remember that, with respect to imaginary elements of the plane, Frege describes the task he set himself as follows: “As in the consideration of points at infinity, there now arises the need, not only for treating these improper elements in the same way as the proper ones, but also for having them before our eyes.” (ibid., 2) Such a “geometrical representation” of imaginary forms is

a kind of correlation in virtue of which every real or imaginary element of the plane has a real, intuitive counterpart. The first advantage to be gained by this is one common to all cases where there is a one-one relation [eindeutige Zuordnung] between two domains of elements: that we can arrive at new truths by a mere transfer [bloße Übertragung] of known propositions. But there is another advantage peculiar to this case: that the non-intuitive relations between imaginary forms are replaced by intuitive ones.^21^

A geometrical representation is thus given by some kind of correlation of the domain to be represented with objects in Euclidean space. However, Frege’s description here is unclear, and perhaps even confused, in several respects. First of all, Frege is ambiguous about the *kind* of correlation that is involved in a representation. The first sentence in this passage insinuates that a geometrical representation is given simply by a function from the domain to be represented to objects in Euclidean space. But in the next sentence Frege suggests that such a function should also be one-to-one, so that different objects are represented by different objects. Second, it is not clear which relations and properties should be preserved in a representation and which ones may be ignored. Both issues are important, since Frege explicitly notes that it is in virtue of one-to-one mappings that we can “transfer” truths about one domain of elements to another. Finally, although it seems clear that the representing objects should be objects of Euclidean space, it is not clear what *kind* of objects are allowed to ‘represent’.

**Fig. 4:**
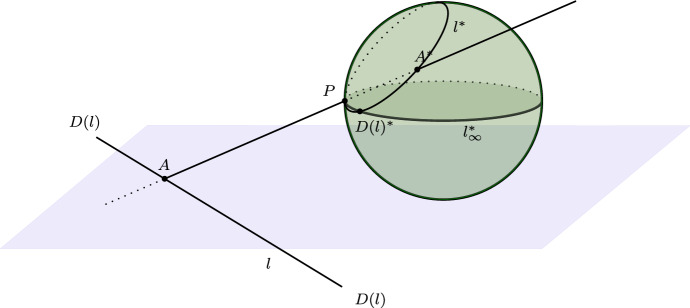
Frege’s ‘geometrical representation’ of points at infinity. A point *A* of the base plane is projected onto the sphere via *P* and represented by its image $A^*$ on the sphere. Lines are represented by circles on the sphere that pass through *P*. The ‘line at infinity’ is represented by a circle $l_{\infty }^*$ on the sphere that lies in the plane through *P* that is parallel to the base plane

That Frege was confused about the first two issues is indicated by an example of an “intuitive representation” he mentions in the introductory section. It is worth looking at this example in a bit more detail. Frege, after setting out the basic idea of his plan of bringing imaginary forms “before our eyes”, says that “this is easily achieved for points at infinity in the plane by projecting the plane on a sphere from a point on the sphere which is neither the nearest nor the furthest” (ibid., 2–3). Frege does not discuss the representation in detail, but only mentions that “in that case there is no difference in projection between proper points and points at infinity” (ibid.).^22^ So the setup Frege likely had in mind is one where we have a plane in three-dimensional space, call it the *base plane*, and a sphere which is placed above the base plane. Call the point on the sphere which is nearest to the base plane the *south pole*, and the one which is furthest from the plane the *north pole*. For the sake of definiteness, let’s assume that the south pole touches the base plane (see Fig. 4). Now, suppose we fix a point *P* on the sphere (our projection center) which is neither the north pole nor the south pole. By connecting a point *A* of the base plane with the projection center *P*, we get another point $A^*$ on the sphere, namely, the second point where the line $\overline{AP}$ meets the sphere. We take this point to be *A*’s representation on the sphere. It is easy to see that a line *l* in the base plane will be projected to a circle on the sphere, namely, the circle $l^*$ that is carved out by the plane that is determined by *l* and *P*. So lines are represented by circles on the sphere that pass through *P*. Furthermore, parallel lines *l* and $l'$ in the base plane will be projected to circles that generally meet in two points, the projection center *P* and an additional point. The latter is then taken as the representation of the common “point at infinity” *D*(*l*) of *l* and $l'$. Finally, all these points lie on the circle that is carved out by the plane through *P* which is parallel to the base plane. Hence, we may take this circle to be a representation of the “line at infinity”.

Now, it is easy to see why Frege would think of this construction as providing a ‘representation’ of elements at infinity. After all, the central property of points at infinity (or directions) is visualized by the mapping: representations of parallel lines meet in a point that we now have “before our eyes”. Just like ordinary points of the base plane, points at infinity are thus represented by ordinary points on the sphere. Also, if two lines in the base plane meet in a certain point *A*, then their representations meet in $A^*$. In modern terms, incidence relations are preserved by Frege’s mapping. However, the example raises some issues in connection with Frege’s general comments about geometrical representations from the passage quoted earlier.

For one, different points of the base plane are not in general mapped to different points on the sphere. In particular, since *P* is assumed to be different from both the north and south pole, the tangent plane to *P* on the sphere will intersect the base plane in a certain line $l_P$. Since the line joining a point *A* on $l_P$ with *P* is tangent to the sphere at *P*, by construction, each point *A* on this line will be mapped to the projection center *P*. Thus, in contradiction to Frege’s general comments about geometrical representations, this particular correlation is not one-to-one. From this observation it also follows that some of the most basic geometrical principles are not satisfied in the structure that is induced by Frege’s mapping. For example, the most fundamental principle of both affine and projective geometry, that two distinct points determine a unique line, is not satisfied in the representation. Any point on the sphere together with *P* determines infinitely many “lines”, i.e., circles on the sphere through *P*. So Frege’s representation is clearly not a model of the real projective plane.^23^ But if not even the most fundamental truths about points and lines must be preserved by a representation, then it is also unclear how the “transfer” that Frege mentions is supposed to work in general. Clearly a “transfer of known propositions” about one domain of elements to another domain is possible only if we can be sure that certain basic propositions are satisfied in both structures.^24^

Putting aside the issue of the precise nature of the mapping that is involved in a representation, there still remains the question of what is required of such a mapping to induce an *intuitive* or *geometrical* representation. As mentioned, Frege thinks that a geometrical representation of a non-intuitive structure is given by a correlation that somehow establishes a correspondence between the non-intuitive forms to be represented and objects in Euclidean space. But what kind of objects in Euclidean space are allowed to occur as correlates?

In his representation of elements at infinity, points and lines are represented by common geometrical objects: points are represented by points on a sphere, and lines are represented by circles on that sphere. But we have seen earlier that Frege is also prepared to consider more complex configurations to do representational duties. Imaginary lines, for example, are represented by *pairs* of Euclidean lines that meet certain constraints, and more complex curves are represented by so-called “guide surfaces” (ibid., 25 ff.). This seems to indicate two things. First, Frege’s early notion of a ‘geometrical object’ was apparently broad enough to include complex configurations that are formed from more commons ones by building pairs and other complexes. Second, since Frege takes himself to provide an *intuitive* representation of imaginary forms, he also seems to be committed to the idea that these complex objects are *intuitive* objects themselves. One problem with this is that it then seems hard to escape the view that all kinds of complexes, including all *classes* of intuitive objects are intuitive objects. Given Frege’s later identification of points at infinity with classes of mutually parallel lines (to be discussed shortly), this is certainly in tension with his expressed view that points at infinity are non-intuitive. True, Frege has not yet made the identification of points at infinity with classes of parallel lines in his dissertation. Still, it can be seen at various places throughout his dissertation that he was wavering with respect to the status of points at infinity.

For example, at some point in his dissertation Frege notes that a certain imaginary straight line can be represented by “a direction or a pencil of parallel lines” (ibid., 36). What is interesting about remarks like these are three things. First, locutions like these already foreshadow Frege’s later identification of points at infinity with classes of parallel lines. Second, they suggest that Frege takes directions to be capable of intuitively representing imaginary forms after all, in contrast to his explicit pronouncement that directions are improper elements that do not belong to the “space of intuition”. Third, the wording in this specific passage seems to indicate that Frege was hesitant to use the term “direction” alone, adding “or pencil of parallel lines”, presumably, to conceal the fact that, in the introduction, he had explicitly rejected the idea that directions belong to the “space of intuition”.^25^ All of this suggests that in his dissertation Frege was still undecided about how to think about elements at infinity, both with respect to their ontological status and with respect to their (non-)intuitiveness.

Another topic that is relevant in the current context relates to representations of the real projective plane as a whole. Around the time Frege was finishing his dissertation, non-orientable surfaces (like the Möbius band) had already been studied by Möbius and Listing, and Klein had shown that the real projective plane is one of them. From these results one could already gather that the projective plane cannot be ‘visualized’ in a straightforward way. It was later shown that, in fact, no closed, non-orientable surface can be topologically embedded in Euclidean space. So representations of the projective plane in intuitive (Euclidean) space are out of the question, since any representation is bound to contain points that represent more than one point of the projective plane. Representations of the real projective plane such as the sphere model or the semi-sphere model mentioned in Sect. 2 therefore obscure the fact that, topologically, the real projective plane is a rather peculiar structure.^26^ I think that Frege, at the time he was writing his dissertation, was not aware of the topological intricacies related to the real projective plane. Indeed, his passing remark that an intuitive representation of points at infinity is “easily achieved” by projection on the sphere points to a somewhat simplistic conception of intuitive representations of the real projective plane. From what we can gather from his early writings, it is not clear that Frege even *had* a conception of the real projective plane as a distinguished structure in its own right. On a charitable reading, then, his example was not intended as a representation of the entire projective plane after all, but merely as a loose way of visualizing elements at infinity ‘locally’.

To sum up, in his dissertation, Frege was ambiguous about several issues related to extension elements, including various aspects related to their ‘geometrical representation’. Still, one should not be too harsh on Frege. For one, Frege was only 25 years old and had just finished his studies. Also, some of the issues we have mentioned, such as topological investigations of the projective plane, were still in their infancy at the time. As we will see though, there is indirect evidence that dissatisfaction with some of his early remarks on extension elements as well as their ‘geometrical representation’ were among the reasons why Frege eventually developed more sophisticated views on mathematical objecthood and intuition in his subsequent writings, especially in the *Grundlagen*. We will come back to this in Sect. 9, but stick to the mathematical period for just a bit longer to see what early Frege has to say on two further issues that were central to nineteenth century projective geometry, the principle of duality and the status of projective geometry vis-à-vis Euclidean geometry.

## The principle of duality

One of the central features of projective geometry is the principle of duality or reciprocity. In the planar case it says that for each theorem we get another theorem by simply interchanging the terms “point” and “line” and, accordingly, the relation of a point lying on a line by the relation of a line passing through a point. (In solid projective geometry the roles of the terms “point” and “plane” are interchanged and the term “line” is self-dual.) To illustrate, take Desargues’ theorem mentioned earlier:

**Table:** 

**Desargues’ Theorem**	**Dual of Desargues’ Theorem**
If corresponding vertices of two triangles lie on three lines that meet in a point, then corresponding sides of the triangles meet in three points that lie on a line.	If corresponding sides of two triangles meet in three points that lie on a line, then corresponding vertices of the triangles lie on three lines that meet in a point.

So the dual of Desargues theorem is its converse and it is a theorem of real projective geometry just like Desargues’ theorem itself.

**Fig. 5:**
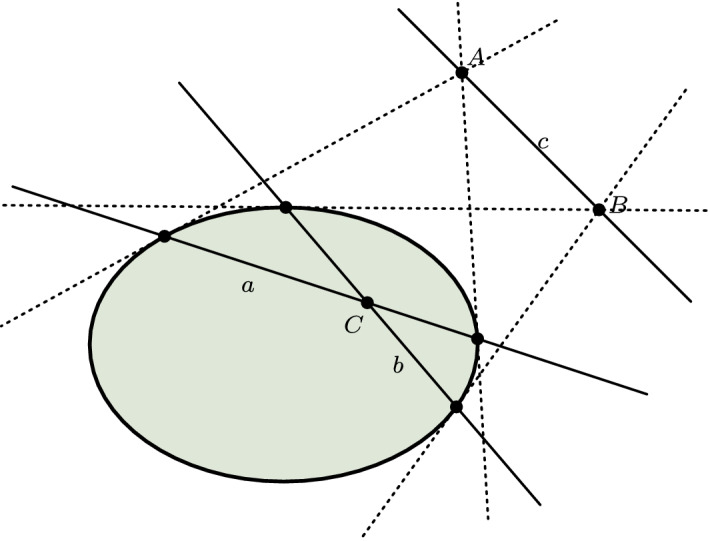
Polar reciprocity with respect to a fixed conic (here, an ellipse). The lines *a*, *b*, *c* are the polars of the points *A*, *B*, *C* respectively, and the points *A*, *B*, *C* are the poles of the lines *a*, *b*, *c*

After the principle of duality was discovered around the turn of the nineteenth century, a bitter controversy between Gergonne and Poncelet ensued over the question of priority, but also about its content and proper justification.^27^ Poncelet thought that duality was a consequence of the theory of poles and polars. The idea is that, with respect to some arbitrary, but fixed, conic section, we can associate with each point of the projective plane a certain line, its polar, and with each line a certain point, its pole. This can be done in such a way that, first, the pole of the polar of a point is the point itself and the polar of the pole of a line is the line itself and, secondly, a point lies on a line just in case the polar of the former passes through the pole of the latter (see Fig. 5). In virtue of this one-to-one correlation between points and lines, each configuration can then be effectively transformed into the dual configuration. Since incidence-properties are preserved by this transformation, theorems about one configuration must correspond to theorems about the dual.^28^ Gergonne, by contrast, thought that duality is not tied to the theory of poles and polars and has nothing specifically to do with conic sections. Instead, he seems to have conceived of duality as a consequence of the symmetric nature of the basic laws of projective geometry (Gergonne 1825/26, 212 ff.). The idea is that for each basic truth of projective geometry there exists another basic truth that is dual to the former. Hence, if we can prove a certain proposition from the basic truths, we can also prove its dual by simply replacing the basic truths used in the proof with their corresponding duals. In order to highlight this symmetry, Gergonne introduced the parallel column format which became standard in nineteenth century presentations of projective geometry and was adopted by many geometers after him. Gergonne’s own presentation contains some shortcomings^29^, but an improved version of Gergonne’s principal conception of duality was eventually developed in Pasch’s famous *Vorlesungen über neuere Geometrie* (Pasch 1882), where Pasch spends the entire section 12 on a detailed discussion of duality.^30^ There is also a third, analytic conception of duality that goes back to Plücker (Plücker 1831). Remember that, on the analytic approach to projective geometry, projective points and lines are represented by means of homogeneous coordinates and the basic incidence-relation between a point (*x* : *y* : *z*) and a line $\left[ u:v:w \right]$ is expressed by the equation

$$\begin{aligned} ux + vy + wz = 0 \end{aligned}$$

Evidently, the contribution of point and line coordinates in this equation is completely symmetrical. So any theorem of analytic projective geometry can be interpreted in one of two ways, depending on whether (*x* : *y* : *z*) and [*u* : *v* : *w*] are interpreted as point- and line-coordinates or as line- and point-coordinates respectively, which is what explains duality according to Plücker.

Now, in one form or another, the principle of duality was recognized to be of central importance and in many treatises and textbooks it received a prominent place right at the beginning. It was also seen as a prime example of a broader class of ‘transfer principles’, i.e., principles that enable a transfer of theorems about one structure to another.^31^ Given its central place in projective geometry, we may ask: How did Frege reflect on duality and which status did it have in his own conception of projective geometry?

Once again, there is no systematic discussion of duality in Frege’s writings. But he does talk about duality in the course of a discussion of projective geometry and its relation to metrical geometry in a review of a textbook on the “newer analytic geometry” (i.e., analytic projective geometry) from 1877. Here, Frege indicates that the principle of duality plays a significant role in his conception of projective geometry. Frege complains that the authors of the textbook would show “insufficient insight into the respective positions of projective and metrical geometry”, and indicates what he thinks is the “true relationship”, noting that “[p]rojective geometry may be likened to a symmetrical figure where every proposition has a proposition corresponding to it according to the principle of duality” (Frege 1984, p. 95). The authors had apparently tried to generalize metrical geometry in such a way that the principle of duality remains valid, introducing concepts like ‘parallel points’ and the like. After complaining about this and noting that to these concepts “nothing corresponds in our intuition”, Frege then says: “To sum it up in brief, the principle of duality can never provide a reason for a specialization of general projective geometry; the consideration of metrical properties can be justified only by the special significance they have for our intuition.” (ibid.) This passage is interesting for several reasons, among them, first, Frege’s reiteration of the idea that Euclidean geometry is closely tied to intuition in a way that projective geometry is not, and, secondly, the idea that somehow projective geometry is ‘more general’ than Euclidean geometry. We will come back to these issues shortly. What is important for the current thread is that the passage also suggests that for Frege the principle of duality is not just one principle among others. Rather, he seems to treat duality almost as something of a defining characteristic of projective geometry.

Given the analytic outlook that is displayed in much of Frege’s early mathematical work, and given the fact that Frege was making use of Plückerian ideas in his own mathematical work^32^, it is reasonable to assume that, at the time, Frege thought about duality in the analytic way suggested by Plücker. But the textual evidence on this is simply too sparse. One reason for this lack of evidence is that Frege had more important things to do. His main interest was in the foundations of arithmetic, not that of (projective) geometry. Also, the principle of duality is a ‘meta-theoretical’ principle that is not immediately about geometrical objects, but about *theorems* about such objects and how they arise from others by systematically replacing certain words by others. At least for the time before his debate with Hilbert, problems of that sort were not at the center of Frege’s attention. As Jamie Tappenden has pointed out, though, there are pieces of evidence that Frege did provide something like an implicit account of duality in the aftermath of his controversy with Hilbert. We will come back to this once we have a better understanding of how Frege’s views further developed during the time between the mathematical and the foundational period. But before that, we will stick to another issue that is raised in the review just quoted, namely, early Frege’s views on the status of projective geometry vis-à-vis ordinary, Euclidean geometry.

## The priority of projective geometry

As highlighted in Sect. 2, projective geometry was considered by many nineteenth century geometers to be a foundation for all of geometry. But the reasons for this view differed among geometers. When projective geometers in the early nineteenth century were attributing some kind of priority to projective over ordinary Euclidean geometry, then this was usually based on two reasons. First, projective geometry is ‘more general’ than ordinary Euclidean geometry because the former is only concerned with (essentially) incidence properties, whereas traditional geometry is also concerned with metrical properties. Secondly, projective geometry is more general in that it contains additional objects besides those of ordinary Euclidean geometry, namely, points and other elements at infinity. As we saw, elements at infinity played an important role in nineteenth century projective geometry. But it is the first contrast, that between projective and *metrical* geometry, which was usually considered to be the more significant one. (Remember the Von Staudt quote from Sect. 2.)

More sophisticated accounts of the relationship between projective and Euclidean geometry emerged over the course of the nineteenth century. A result by Arthur Cayley was critical in this respect. Cayley had shown that various metrics can be defined in an analytic setting in purely projective terms by fixing a certain quadric, called the ‘absolute’, as a standard of reference. By considering a particular quadric corresponding to a certain degenerate conic, we can then recover the ordinary, Euclidean notion of distance. It was for this specific reason that Cayley had thought about projective geometry as prior to Euclidean geometry, and it was this discovery that led to his slogan that “descriptive geometry is *all* geometry and reciprocally” Cayley (1859, p. 90). Cayley’s point of view was later extended by Felix Klein to cover non-Euclidean geometries as well. Based on these results, Klein developed a general way of conceptualizing the relative positions of various geometries in his famous ‘Erlangen Program’ from 1872. According to this point of view, a geometry is characterized by a certain *group of transformations*. Projective geometry, for example, is characterized by the group of *projective transformations* or *collineations*, i.e., the group of all the bijective mappings of the projective plane to itself that leave projective properties (such as incidence, separation of point pairs, cross ratio, etc.) fixed. Given Cayley’s result, metrical properties can be understood as those properties that are invariant under all projective transformations that leave the ‘absolute’ fixed. So this gives us a nice way to think about the relative generality of projective over metrical Euclidean geometry: Projective geometry is more general than (and in this sense ‘prior to’) metrical Euclidean geometry because the metrical transformations simply appear as a *subgroup* of the projective transformations.^33^ At the time, Klein was not as famous as he would eventually become, but Cayley’s result was well-known by the 1870s. So it seems safe to assume that Frege was aware of these developments. The question, then, is: How, if at all, did they affect his understanding of the relationship between projective and Euclidean geometry?

First of all, while elements at infinity may have been relevant for his conception of the projective point of view, just like for most nineteenth century geometers, the really important contrast for Frege is that between projective geometry as an inherently *non-metrical* geometry and Euclidean geometry as an inherently *metrical* geometry. This is already indicated in his dissertation (Frege 1984, p. 3), and it is reiterated more explicitly in the review from 1877 mentioned earlier, where Frege says that metrical geometry arises by “specialization” from projective geometry. So what exactly does that mean? Frege gives an indication of his view in the context of a discussion of trilinear coordinates, which are a particular kind of homogeneous coordinates. In the course of this discussion Frege notes that these coordinates are “most intimately connected with the most general relationship, that of collineation, in virtue of which any point has a point and any straight line a straight line corresponding to it” (Frege 1984, p. 96). Projective properties are then identified with those properties that are “preserved under a collinear projection” (ibid.).^34^ Frege does not explain his view in detail, but the basic idea seems to be clear enough: Projective and metrical geometry can be characterized in terms of their characteristic transformations, just like on Klein’s account. Projective geometry results if we consider all collineations, and metrical geometry arises by restricting ourselves to specific collineations that preserve additional properties. It is in this sense that metrical geometry “arises by specialization” from projective geometry. However, even though transformations seem to play a central role in this account, it is worth emphasizing that this is not really the Kleinian point of view.

First of all, Klein intended to provide something of a general classification scheme for all the important geometries of the time. By contrast, Frege’s remarks really only mention projective and Euclidean geometry. So it is not clear how other geometries are supposed to fit into the picture. Secondly, unlike Klein, Frege does not specifically mention the notion of a *transformation group*, which is one of the key ideas in Klein’s account. Finally, Klein’s central message (the priority of projective geometry) was closely linked to Cayley’s result, which, in the hands of Klein, becomes the idea that metrical properties are those that are invariant under projective transformations that leave the ‘absolute’ fixed. But this specific idea does not seem to play any role in Frege’s account. It is not even mentioned. So either Frege wasn’t familiar with Cayley’s results after all, or he did not put the same weight on them as other geometers, such as Klein.^35^ Frege’s view thus seems to be merely a more ‘modern’ expression of the traditional idea, emphasized by many geometers before Klein, that projective geometry is concerned with properties that are unaltered by projection.

Let’s sum up our discussion so far. First, early Frege’s remarks on the relationship between projective and metrical geometry suggest that he thought about their difference informally in terms of transformations, and not, say, in terms of consequences of a certain set of basic truths or ‘axioms’. Despite his early talk about geometrical axioms in his dissertation and elsewhere, projective geometry is not yet identified with the theory of a particular structure, namely, real projective space. Instead, Frege seems to believe that projective geometry deals with ordinary, Euclidean space just like Euclidean geometry. It is just that both geometries focus on different aspects of this particular structure. Euclidean geometry is concerned with certain transformations and the properties that are preserved by them, while projective geometry is concerned with a more encompassing class of transformations. Talk about elements at infinity is useful in this context because it enables us to conduct our investigations in a perspicuous and unified manner. On the other hand, while Frege sometimes seems to characterize talk about points at infinity as a useful *façon de parler*, at other times he seems to attribute a more robust ontological status to points at infinity. Early Frege does not give us a clear account of what exactly elements at infinity *are*, and how we can have epistemic access to these entities, which he maintains are non-intuitive in character. In the following sections we will look at how Frege’s views on these and related matters develop further.

## Elements at infinity in the *Grundlagen*

A new period in Frege’s thinking about geometry begins sometime between the *Begriffsschrift* in 1879 and the *Grundlagen* from 1884. Generally speaking, this period is characterized by two developments in Frege’s thinking. First, as a result of becoming more serious about his logicism, Frege had become more interested in logical matters and developed his first logical system, the Begriffsschrift. Secondly, this new phase is marked by a substantial increase in philosophical considerations that were intended to give his logicist thesis a sound philosophical foundation. Frege had expressed his belief in a fundamental difference between arithmetic and geometry already in his habilitation dissertation (Frege 1984, pp. 56–57). But it is only in the *Grundlagen* that he is trying to flesh out such vague allusions. For now, we stick to the question how these new philosophical considerations might have affected his views on elements at infinity, and how, conversely, they might have been nurtured by concrete mathematical concerns about those elements.

As we saw, in his early mathematical writings, Frege was ambiguous about the nature of elements at infinity. A clearer view on this is developed in the *Grundlagen*, where Frege spends several paragraphs on the discussion of points at infinity without even mentioning the term “point at infinity”. Instead, he is now consistently using the term “direction”. Here are the main points that are relevant for our main thread, many of which have been discussed in the excellent (Mancosu 2015, 2016).

As is well-known, Frege’s main concern in the *Grundlagen* is to give an outline of his logicist project of reducing arithmetic to logic. Part of this project, in his view, involves showing how terms for individual cardinal numbers can be introduced in such a way that these expressions can flank the identity sign, and, thus, function as designating expressions that refer to particular objects. At a certain juncture, he contemplates doing this by means of a *principle of numerical abstraction* (nowadays known as *Hume’s principle*) which says that the number of *F*’s is identical with the number of *G*’s just in case the *F*’s and *G*’s are equinumerous. Here, the expression “the number of” is a term-forming operator that attaches to concept-terms to form singular terms, and equinumerosity is an equivalence relation between concepts that is defined in terms of bijective mappings between concepts. Before addressing the case of cardinal numbers in more detail, Frege first considers various similar principles in the paragraphs 64–68, all of them related to geometry. He focuses on one principle in particular, namely the one that relates to directions. In the course of this discussion, Frege explicitly acknowledges the object-status of directions, noting that “the direction of *a* plays the part of an object” (Frege 1884, p. 77). Frege also reiterates his claim from the doctoral dissertation that directions are non-intuitive, now adding more specifically that “the concept of direction is only discovered at all as a result of a process of intellectual activity which takes its start from intuition” (ibid., 75).

So what does Frege mean by this “process of intellectual activity” and how does it lead to the concept of a direction? In analogy to his approach to cardinal numbers, Frege’s initial idea is to introduce directions in terms of a principle which we might call the *principle of directional abstraction* (DA). It says that for all lines *f* and *g*, the direction of *f* is identical with the direction of *g* just in case *f* and *g* are parallel. So, just as in the case of cardinal numbers, we have again a term-forming operator “the direction of” and an equivalence relation between straight lines, the relation of parallelism, which is a relation between ordinary lines, and, thus, ‘intuitive’. After some back-and-forth, Frege eventually rejects the idea of introducing directions by means of DA *alone* because of what came to be known as the “Julius-Caesar problem”.^36^ Frege’s way out of this conundrum is well-known and is described in §68. Instead of conceiving of DA alone as justifying the introduction of directions, Frege takes DA to be something like an adequacy condition for an appropriate definition of the direction-operator. Similar to the case of cardinal numbers and the number-of-operator, an explicit definition of the direction-operator and directions is then given in terms of classes of parallel Euclidean lines. That is, the direction of a line *l* is defined as the class of all Euclidean straight lines that are parallel to *l*. This seems to have been his position at least until 1905, where the same view is mentioned in the correspondence between Frege and Pasch.^37^

So what is new in Frege’s approach to elements at infinity in the *Grundlagen* compared to his earlier views? As we have mentioned, generally speaking, Frege became much more deliberate about broadly logical matters, in particular, about the distinction between concept and object. One of the guiding principles of the *Grundlagen*, remember, is “never to lose sight of the distinction between concept and object” (ibid., xxii). This clearly affected his views on elements at infinity. First of all, points at infinity are not only acknowledged to be entities of some sort, but *objects* (as opposed to concepts). In the *Grundlagen*, Frege cites a quite specific reason for this, namely, because they are given to us in the form of expressions “the direction of *l*”. Given Frege’s well-known doctrine that the definite article indicates objecthood, we are thus forced to acknowledge the status of directions as objects (Frege 1884, p. 77, fn. 2). But apart from philosophical considerations, mathematical reasons too seem to have led Frege to conceive of directions as genuine objects. To illustrate, take, for example, Frege’s geometrical representation of points at infinity in terms of a projection on the sphere. If we want to think of such a projection as a uniform mapping, then we need a well-defined set of entities which forms the domain of the mapping. But that is possible in a straightforward way only if we treat elements at infinity as being on par with ordinary points and lines. In general, all kinds of issues in projective geometry that involve functional relations require that we take elements at infinity to be genuine objects which we can operate with just as we can operate with ordinary points. After all, it is hard to see how we should conceive of such mappings if, strictly speaking, talk about elements at infinity is merely a *façon de parler*.^38^ So there seem to be different reasons why Frege feels the need to treat points at infinity as genuine objects, some more ‘philosophical’, and some more ‘mathematical’.

But in the *Grundlagen* Frege not only acknowledges the status of points at infinity as genuine objects, but he introduces them as objects of a special kind, namely, *classes* (or extensions of concepts). In fact, he discovered a general method for defining all kinds of mathematical objects like *positions*, *shapes*, and, of course, *cardinal numbers*.^39^ Given this general method, all of these objects can now be *reduced* to classes of objects whose presence can be taken for granted, provided only that an identity criterion can been found in the form of a suitable equivalence relation on the given objects. As we know, Frege’s strategy of reducing mathematical objects to classes proved to be fatal, because it forced him to make his inconsistent theory of classes (or extensions) explicit. It is all the more interesting to see that in the case of points at infinity Frege stuck to this conception even after he received Russell’s letter, as is witnessed by his correspondence with Pasch (Frege 1980, p. 106). We don’t know for sure whether he continued to hang on to this conception because he hoped that parts of his inconsistent theory of extensions could be salvaged after all, or whether he believed that it could be replaced by something else that could do its job instead. In any case, it is remarkable that even in the face of inconsistent foundations, Frege stuck to his approach to points at infinity, which clearly indicates his deep commitment to this conception.

## A new perspective on projective geometry

So what does Frege’s new approach to elements at infinity tell us about his conception of projective geometry? Once again, Frege doesn’t explicitly discuss this issue in the *Grundlagen* or elsewhere. However, a reasonable reconstruction of his view may be extrapolated from his treatment of arithmetic and his analogous definition of numbers in the *Grundlagen*. We will restrict ourselves to the planar case.

First of all, now that elements at infinity are explicitly acknowledged to be definite objects to operate with, we can think of the real projective plane as a definite structure that is obtained by extending the Euclidean plane. To distinguish the two structures, we will refer to Euclidean objects as E-points, E-lines and the basic relation between these objects as E-incidence. Analogously, we will refer to the elements of the extended structure as P-points, P-lines, and P-incidence. Starting with the ordinary Euclidean plane, we can construct the real projective plane as follows. First, we stipulate that the P-points consist of all E-points plus all directions, i.e., all sets of mutually parallel E-lines. The P-lines consist of all E-lines where to each E-line we add its associated direction, and, in addition, there is a “line at infinity”, which may be identified with the set of all directions. We then stipulate that a P-point *A* is P-incident with a P-line *l* just in case *A* is an E-point, *l* is an E-line and *A* is E-incident with *l* *or* *A* is a direction, *l* is an E-line and *l* is an element of *A* *or* *A* is a direction and *l* is the line at infinity.^40^

In analogy to the case of arithmetic, it is easy to derive the principle of directional abstraction DP from these definitions, which ensures their adequacy. Then, using the axioms of Euclidean geometry as a basis, it is straightforward to derive principles like the following:

- (P1) *If* *A* *and* *B* *are distinct P-points, then there is exactly one P-line* *a* *that is P-incident with* *A* *and* *B*.
- (P2) *If* *a* *and* *b* *are distinct P-lines, then there is exactly one P-point* *A* *that is P-incident with a and b*.

Propositions (P1) and (P2) are the two most fundamental principles of projective geometry, the existence and uniqueness of joining lines for any two P-points, and the existence and uniqueness of intersection points for any two P-lines.^41^ Further basic principles of projective geometry may be derived in a similar way, using the axioms of Euclidean geometry and definitions. Also, further notions for projective invariants (e.g., separation of point pairs) can be introduced by explicit definition and basic principles can be proved about these notions, again, based on the axioms of Euclidean geometry. In this way, one eventually arrives at a system of basic principles that suffices for all of projective geometry. Note again the analogy with Frege’s treatment of arithmetic. Just as the basic laws of arithmetic are shown to be reducible to logic plus definitions, on the reconstruction proposed here, projective geometry is considered to be reducible to Euclidean geometry plus definitions. In his (Wilson 2010), Mark Wilson has coined the apt term “relative logicism” for this kind of approach to extension elements in geometry. Importantly, following this approach, projective geometry becomes a theory about a particular structure, namely, the Extended Euclidean plane.^42^

Now, if this is a fair reconstruction of Frege’s views in the *Grundlagen*, then projective geometry seems to become much more of a deductive discipline compared to the early, mathematical period. While projective geometry may still be characterized informally in terms of transformations and the properties that are preserved by them, when it comes to projective geometry as a *systematic discipline*, the crucial thing is to look at what its basic concepts and truths are. Also, since projective geometry now appears as a definitional extension of Euclidean geometry, Euclidean geometry becomes *prior* to projective geometry in two important respects. First, Euclidean geometry is ontologically prior to projective geometry because the objects of projective geometry are defined in terms of Euclidean objects. Second, Euclidean geometry is epistemically prior to projective geometry because its basic truths are ultimately derived from the axioms of Euclidean geometry. Knowledge of projective geometry thus depends on prior knowledge of Euclidean geometry.

It is well-known that throughout his career Frege’s thinking was dominated by a call for rigour that was oriented towards gapless proofs and explicit definitions. In some of his later writings Frege also becomes more outspoken about the nature of mathematics as a systematic endeavour that is ultimately based on primitive concepts and basic truths or ‘axioms’.^43^ It is true that Frege was critical of certain conceptions of the systematic nature of mathematics proposed by contemporaries (see his controversy with Hilbert on the nature of ‘axioms’). But, generally speaking, he certainly welcomed the development towards axiomatization in geometry and mathematics in general. So even though a lot is left open by Frege’s scattered remarks and there is only indirect evidence for the reconstruction proposed here, it seems to me that it blends nicely with his general intellectual outlook.

## The principle of duality revisited

If the reconstruction of Frege’s conception of projective geometry from the previous section is on the right track (projective geometry as a deductive discipline that is derived from Euclidean geometry plus definitions), then we also get a relatively clear picture of how Frege likely conceived of the content and justification of the principle of duality. The prediction would be that duality would be cashed out by Frege along the following lines.

Suppose we start with some system of axioms for ordinary, Euclidean geometry. From these we can derive basic propositions about P-points, P-lines and P-incidence in the way indicated above. As it turns out, we can do this in such a way that for each basic proposition P we can also derive another proposition P$^{\text {D}}$ that results from the former by interchanging the terms ‘P-point’ and ‘P-line’, just as we can interchange these words in the basic propositions (P1) and (P2). But then, since basic principles always come in pairs, the planar principle of duality can be justified simply by pointing to the symmetric nature of the basic propositions. Assuming that each theorem of projective geometry can be deduced from these basic principles, to prove the dual of a theorem P we simply modify the proof of P so that instead of the basic propositions used in the proof of P, we now use their duals. Making use of the fact that deductive proof is *formal* in the sense that it remains valid when we uniformly substitute non-logical terms by others, the principle of duality then follows.

Although there is no evidence from the time of the *Grundlagen* that Frege explicitly endorsed such a conception of duality, as Jamie Tappenden has first pointed out in his Tappenden (2005), there is indirect evidence for such a view in a later piece. In a series of articles titled “Die Grundlagen der Geometrie”, published between 1903 and 1906, Frege presents in detail his critique of Hilbert’s views on axioms, definitions, and, more specifically, independence proofs in geometry.^44^ In the last part of the 1906 paper, he presents his own account of how to prove independence. In brief, Frege suggests that in order to prove the independence of a proposition P from a group of axioms A, we first have to find a certain ‘translation manual’ that allows us to translate propositions into other propositions according to the manual. He then asks the reader to imagine two parallel columns where to each proposition in the first column there corresponds a proposition in the second column that arises from the first by substituting non-logical terms according to the translation manual. In a similar way, we can also consider entire sequences of propositions in both columns. Now, because of the “formal nature of logical laws” (Frege 1984, p. 337), to every valid proof in the first column there must then correspond a valid proof in the second. So if we can come up with a translation manual that allows us to translate the axioms in A into a group of *true* propositions A$^{\text {T}}$ and P into a *false* proposition P$^{\text {T}}$, then P must be logically independent of A, because if P were provable from A, then, by the the formal nature of logical laws, P$^{\text {T}}$ would have to be provable from A$^{\text {T}}$, which, however, is impossible since only true propositions can be provable from true propositions.^45^

Although Frege does not explicitly mention duality in the context of his presentation, Tappenden is certainly right that it is highly unlikely that Frege wrote this passage without the principle of duality in mind. I also agree with Tappenden that it is “inconceivable that Frege would not have expected the readers of the mathematics journal in which this essay appeared to see the connection automatically” (Tappenden 2005, p. 214). If this is correct, then Frege’s explanations can be understood as an implicit account of duality, where duality is conceived as a principle that is based on dual deductive proofs from dual basic truths or ‘axioms’. Specifically, by the “formal nature of logical laws”, it follows that whenever a proposition P can be proved from a set of basic propositions A that is assumed to be symmetrical (i.e., for which A = A$^{\text {D}}$), then its dual P$^{\text {D}}$ must be provable as well. As we have seen, this is essentially the way in which Gergonnne and later Pasch had thought about duality (in contrast to both the transformation-based conception of duality in the spirit of Poncelet and to the analytic conception favoured by Plücker).^46^ Now, while there is no hard evidence that Frege read Pasch’s *Vorlesungen* at the time he was writing the *Grundlagen*, he almost certainly read them around the time he was working on the second series on the foundations of geometry, when he was also corresponding with Pasch. So my conjecture is that Pasch’s extensive discussion of duality and of the formal nature of deductive proof in connection with duality in §12 of the *Vorlesungen* might have been a key influence for Frege’s 1906 proposal concerning independence proofs.^47^

## Projective geometry and intuition

In the foregoing sections we have been talking a lot about ‘intuitive representations’ and objects being ‘(non-)intuitive’. It is now time to say more on Frege’s conception of intuition. In the very first sentence of his first published work, his doctoral dissertation, Frege states that “the whole of geometry rests ultimately on axioms which derive their validity from the nature of our intuitive faculty” (Frege 1984, p. 1). A similar point is made a year later in his habilitation dissertation, where Frege identifies an important difference between arithmetic and geometry, noting that, in contrast to arithmetic, “the elements of all geometrical constructions are intuitions, and geometry refers to intuition [Anschauung] as the source of its axioms” (ibid., 56–57). As we saw in Sect. 7, similar remarks are made in the context of a discussion of duality. So what are Frege’s views on intuition and how do they square with his views on projective geometry?

First of all, even though Frege took courses on philosophy, it is not clear how seriously we should take his early references to intuition, and whether they were intended as a commitment to any specific doctrines, especially Kantian ones.^48^ Remember that Frege was only at the beginning of his career. Also, generic references to intuition as the source of the axioms of geometry were not uncommon in the works of German geometers throughout the nineteenth century. Even Hilbert, who is not usually suspected of being a transcendental idealist, notes in his famous *Grundlagen der Geometrie* from 1899 that the axioms of geometry express “basic facts of our intuition” (Hilbert 1899, p. 2). A specifically Kantian influence on Frege can be made out, however, by the time of the *Grundlagen*. The extent of Kant’s influence on Frege is a delicate issue and several commentators have written on this topic.^49^ Generally speaking, Kant plays a role in the *Grundlagen* in at least two respects. First, more than in any other of his writings, Frege is relying on Kantian terminology. He refers to Kant’s distinction between synthetic and analytic judgements and he frequently uses the term ‘intuition’. Secondly, Kant’s views on the status of the truths of arithmetic and that of geometry are used by Frege as the main point of reference for his own views. While Kant is praised for revealing the “true nature” of the truths of geometry by calling them synthetic a priori, he is criticized for putting the truths of arithmetic into the same category (Frege 1884, pp. 101–102).

With respect to the first point, remember that Frege reformulates Kant’s distinction between synthetic and analytic judgements in light of his new, more fine-grained analysis of the logical structure of judgements. While this leads to important differences in their conceptions of the analytic-synthetic distinction, a lot of agreement remains.^50^ The situation becomes more difficult when it comes to Frege’s and Kant’s conceptions of intuition. Generally speaking, Frege and Kant are in agreement that geometry is not grounded in experience (Frege 1980, pp. 114–24) and that the truths of geometry are synthetic a priori and based on intuition. However, there are significant differences in their views about what exactly intuition *is* and *how* it is supposed to ground geometry. For example, Kant explicitly *argues* that the acquisition of geometrical knowledge requires intuition because logical analysis of geometrical concepts alone “will never give us anything new” (Kant, 1781/1787, p. 632). By contrast, Frege seems to take it for granted that geometry is grounded in intuition and nowhere *argues* for such a claim. A further difference is that Kant thinks that intuition guides the mathematician in geometrical *proofs*^51^, whereas Frege likely wants to limit the role of intuition to explaining how we have knowledge of the *axioms* of geometry. According to Frege, a mathematical proof is a sequence of propositions, where each step has to be in accordance with a law. This is true for geometrical proofs just as it is true for arithmetical proofs. It is just that in the former case we have to use certain laws that are specific to geometry and which cannot themselves be derived from purely logical laws. Intuition justifies our taking the axioms to be true, but it has no further justificatory role to play in geometrical proofs.

Differences like the ones just mentioned are significant, to be sure. But there are even more severe differences between Kant and Frege when it comes to the notion of intuition. The most extensive discussions of intuition in the *Grundlagen* (and Frege’s entire oeuvre for that matter) can be found in §§12 – 14, where Frege discusses two different conceptions of intuition he finds in Kant^52^, and then again in §26 in a passage that eventually leads to a discussion of objectivity:

Space, according to Kant, belongs to appearance. For other rational beings it might take some form quite different from that in which we know it. Indeed, we cannot even know whether it appears the same to one man as to another; for we cannot, in order to compare them, lay one man’s intuition of space beside the other’s. Yet there is something objective in it all the same; everyone recognizes the same geometrical axioms, even if only by his behaviour, and must do so if he is to find his way about the world. What is objective in it is what is subject to laws, what can be conceived and judged, what is expressible in words. What is purely intuitable is not communicable. (Frege 1884, 35)

Despite Frege’s reference to Kant in this passage, it seems to me that, maybe with the exception of the first two sentences, none of this seems particularly Kantian. Kant certainly *did* think that space appears the same for everyone, because space, like time, is an *a priori* intuition that arises “by fixed law from the nature of the mind” (Kant 1894, p. 65). Also, whether Kantian or not, Frege’s reasoning is not very convincing. Frege makes clear elsewhere that by “axioms of geometry” he means the axioms of *Euclidean* geometry. However, it is not at all obvious why we should have to accept the axioms of Euclidean geometry in order to “find our way about the world”. After all, one might reject Euclidean geometry on a large scale, and yet accept that, locally, space is approximated by Euclidean geometry.^53^

Frege’s remarks indicate a deep disagreement with Kant on some of the fundamental features of the concept of ‘intuition’, whether space is subjective or objective, and how exactly intuition is supposed to ground geometry. Frege claims that we cannot be sure that space appears the same for everyone because we cannot compare one person’s intuition of space with that of another. This kind of argument is used by Frege in other contexts as well to show that certain kinds of entities are purely subjective and therefore belong to psychology.^54^ Indeed, this seems to be precisely how he conceives of intuitions. As he says: “What is purely intuitable is not communicable.” He further clarifies that “I understand objective to mean what is independent of our sensation, intuition [Anschauen] and imagination, and of all construction of inner pictures out of memories of earlier sensations” (Frege 1884, p. 36). So intuitions are assimilated to inner pictures and other purely subjective entities that are explicitly acknowledged to belong to psychology by Frege. It is true that for Kant, too, space is subjective. But when Kant is using the term “subjective” he is not using it in its *psychological* sense. Rather, what is subjective is what is common to *all* subjects. Space is subjective in that it is a feature of the human mind in general, it is a condition for the possibility of experience, and, thus, *a priori*. By contrast, at least in the *Grundlagen*, Frege *does* seem to understand the notion of intuition in the psychological sense.^55^

The issue is complicated even further by Frege’s discussion of a thought experiment involving the solid version of duality that succeeds the passage just quoted. Remember, the solid principle of duality states that for each theorem of solid projective geometry there is another theorem that arises from the first by interchanging the terms “point” and “plane”. Frege’s thought experiment then runs as follows: Consider two rational beings that can only intuit projective relationships and suppose that what one intuits as a point, the other intuits as a line and vice versa. Since all theorems of projective geometry are dual, “over all geometrical theorems they would be in complete agreement, only interpreting the words differently in terms of their respective intuitions” (Frege 1884, 36). Frege infers from this that *if* the meaning of a geometrical term *were* the intuition corresponding to it, then, since “what is purely intuitable is not communicable”, geometrical terms would have no objective meaning, which he apparently considers to be absurd. Therefore, Frege’s conclusion is that the meaning of a geometrical term must not be identified with the intuition that corresponds to it after all. Now, remarks like these open up an entirely new set of further questions that are related to Frege’s conception of meaning, which I cannot discuss here in detail. What is important for our current thread is that if we take his remarks at face value, then it seems that we have no reason to believe that the axioms of geometry are ‘grounded’ in intuition in a way that would account for their apparent objectivity and necessity.^56^

I don’t think that there is a clear way out of this conundrum based on anything Frege says in the *Grundlagen*, or elsewhere for that matter.^57^ It seems to me that, despite his references to Kant, Frege did not subscribe to any specifically Kantian doctrines on geometry in the way they were intended by Kant after all. Instead, in the *Grundlagen* he was using Kantian themes in a loose way to flesh out his logicist project philosophically in order to make it more appealing to a broader audience. In any case, the textual evidence suggests that by ‘intuition’ Frege refers to some kind of inner impression that is understood on the model of ordinary visual perception, and, thus, the ‘faculty of intuition’ seems to reduce to some psychological ability after all.

Where does that leave us with respect to Frege’s views on non-intuitive elements in geometry? First of all, whatever Frege’s conception of intuition might have been, we do get a relatively straightforward picture of how elements at infinity fit into the picture once we have settled on *some* conception. Remember, in the *Grundlagen* Frege says that “the concept of direction is only discovered at all as a result of a process of intellectual activity which takes its start from intuition”. So the general idea seems to be this: Proper geometrical objects are objects whose existence is entailed by the axioms of Euclidean geometry, which is about Euclidean space, “the only space of which we have an intuition” (Frege 1884, p. 20). Thus, being an intuitive object just *means* being an object of Euclidean space. Improper geometrical objects, like elements at infinity or imaginary elements, do not belong to the “space of intuition” because their existence is ruled out by the axioms of Euclidean geometry. However, we can nonetheless start from the axioms of Euclidean geometry and introduce such objects by definition in the way indicated earlier. And while these objects are not intuitive themselves, they are nonetheless *grounded* in intuition.

Now, the idea that intuitive geometry simply *is* Euclidean geometry was certainly not universally accepted among nineteenth century geometers. Von Staudt and other projective geometers in the synthetic tradition were often seen as doing ‘intuitive’ geometry.^58^ And it is easy to understand why one would think that. Nineteenth century geometers in the synthetic tradition were typically thinking of the real projective plane in terms of something like the disk-model mentioned in Sect. 2, where the Euclidean plane is represented by the interior of an ordinary Euclidean disk, and the ‘line at infinity’ is represented by its boundary circle (cf.  Fig. 1, left). Once such a picture of the projective plane is adopted, many facts about the projective plane appear to be ‘intuitively obvious’. For example, projective straight lines can be ‘seen’ to be closed, circle-like curves (although we have to intuit antipodal points on the boundary circle ‘as one’). Conic sections, including the hyperbola and the parabola, can be ‘seen’ to be closed curves as well, and it can be ‘seen’ that they divide the plane in two separate regions, whereas projective straight lines do not. Frege would agree that all of this is ‘intuitive’. But I think that Frege would insist that, strictly speaking, this intuitiveness must be attributed to the *representation* of the projective plane, in this case, the disk-model, not the projective plane itself. I think that remarks like the following, although aimed at non-Euclidean geometries, also apply to projective geometry:

To study such conceptions is not useless by any means; but it is to leave the ground of intuition entirely behind. If we do make use of intuition even here, as an aid, it is still the same old intuition of Euclidean space, the only space of which we have an intuition. Only then the intuition is not taken at its face value, but as symbolic of something else; for example, we call straight line or plane what we actually intuit as curved (Frege 1884, p. 20).^59^

Again, I think that Frege believes that something similar can be said about projective geometry as understood by him. Representations of the projective plane like the disk-model or the sphere model are useful because they give us a picture that can be used “as an aid” to discover truths about the projective plane. But while in this way truths about the intuitive representation can be transferred to the projective plane, the projective plane itself must not be *identified* with any of these representations. Rather, as we have seen in Sects. 9 and 10, the real projective plane is a non-intuitive structure (because partly about non-intuitive objects) that arises from the Euclidean plane by definitional extension.

## Concluding remarks

Let me summarize again the main points of the previous sections. We have seen that some of the central themes of Frege’s thinking about geometry are already present in his early mathematical works. Among them are some general themes, such as Frege’s emphasis on the fundamental difference between arithmetic and geometry and the idea that geometry is based on intuition, and, on the other hand, more specific views, such as the idea that elements at infinity are “improper” objects that do not belong to intuitive space. Frege’s views on these matters become more determinate, partly for mathematical reasons and partly for philosophical reasons, during the period leading up the *Grundlagen*, where Frege settles for a definite view on elements at infinity and the real projective plane as a structure that arises by extending the real Euclidean plane. We have reconstructed Frege’s conception of projective geometry during this period in terms of the theory of this particular structure, and noted that this is in tension with his informal, transformation-based view from earlier. Since, according to the new picture, projective geometry is a theory about a particular structure that arises from Euclidean geometry by definitional extension, we also get a picture of how projective geometry, although in part about non-intuitive objects, is nonetheless *grounded* in intuition. In this way, we were able to reconstruct a story about how projective geometry fits into Frege’s conception of geometry, in which Euclidean geometry still takes center stage. But we also said in Sect. 2 that projective geometry was not the only important ‘new’ geometry that came up during the nineteenth century geometry. So what about all the other geometries? How do they fit into Frege’s general conception of geometry?

I have to leave a detailed answer to this question for another paper. But let me mention a couple of important points. First of all, I think that even the most careful reading of Frege’s writings will not reveal a conception of what ‘a geometry’ is beyond what was *regarded* at the time as ‘a geometry’. This is in contrast to other major figures in late nineteenth century geometry. Klein’s ‘Erlangen Program’ can be understood as one way to systematize the multitude of geometries that emerged during the nineteenth century. Hilbert’s formal axiomatic approach, where axioms are no longer tied to a fixed interpretation and can be combined in various ways, was another.^60^ Frege had no such general vantage point that would have enabled him to classify and compare all the different geometries that were emerging at the time. Instead, he seems to have rationalized some of them in terms of ordinary Euclidean geometry, and others in some other way. To illustrate, take, for example, geometries of more than three dimensions that arise by conceiving of the parameters in equations as coordinates in the style of Plücker. As Frege notes in his “Lecture on Pairs of Points in the Plane” from 1884, we can study geometries of this kind “without leaving the firm ground of intuition” Frege (1984, p. 103). So apparently Frege thought that by engaging in these kinds of investigations we still study ordinary, Euclidean space. It is just that, in these cases, space is ‘carved up’ (a term used by Mark Wilson) in a non-standard way and different ‘space elements’ are taken to be fundamental. A geometry like Frege’s own geometry of pairs of points in the plane, for example, may be four-dimensional and carve up the plane in a non-standard way (pairs of points, considered as “fused unities” Wilson (2010, p. 402) are taken as primitive), yet it is about the Euclidean plane all the same.^61^

But this kind of ‘reduction’ does not obviously work in all cases. In particular, it seems that non-Euclidean geometries cannot be rationalized along these lines in a straightforward way. Although Frege’s views on such genuine alternatives to Euclidean geometry are not entirely consistent over time, something of an account of how to understand such geometries emerges in the course of his controversy with Hilbert. Basically, the idea is to conceive of non-Euclidean geometries in terms of *higher-order concepts* that result from genuine propositions by replacing the primitive geometrical terms by (higher-order) *variables*. We can then investigate the logical relations between such ‘axiom schemas’, and we can also study particular structures in which groups of axiom schemas are ‘satisfied’.^62^ Although Frege’s rhetoric with respect to non-Euclidean geometries became more radical in some of his later writings, even then he seems to be open to the idea that alternative geometries can be meaningfully investigated by conceiving of them as higher-order concepts.^63^

To sum up, although much is left implicit in Frege’s writings, an account of projective geometry and its relation to ordinary, Euclidean geometry can be extracted from them. This account has clear points of contact with conceptions in both nineteenth century and contemporary mathematics. However, its philosophical motivation is old-fashioned (even for Frege’s time) and its methodological outlook is limited. Frege seems to have had no general conception of geometry that would enable him to deal with the diversity of geometrical research during the nineteenth century in a unified way. Again, at least in part, this is due to his conservative views about geometry, where geometry is understood as the theory of a particular structure, namely *Euclidean space*, which is somehow given to us by ‘intuition’. Frege was not alone with such views, and he could not have foreseen the significance that, say, non-Euclidean geometries would eventually gain for our understanding of physical time and space. Still, I think it is fair to say that one would expect a more sophisticated conception of the foundations of (projective) geometry from someone as creative as Frege. Then again, Frege’s contributions to logic and its philosophy are monumental and there is only so much a single person can achieve.

## Data Availability

not applicable

## Footnotes

1 See Blanchette (2012), Mancosu (2015, 2016), Schirn (2019), Shipley (2015), Tappenden (2000, 2005, 2006), Wilson (1992, 2005, 2010). See also Eder (2019).

2 Developments in nineteenth century geometry are discussed in (Freudenthal 1962; Nagel 1939; Gray 2007; Kline 1972; Torretti 1978). For an overview of projective geometry in the nineteenth century, see Coolidge (1934). Modern presentations of projective geometry can be found in Coxeter (1987), Richter-Gebert (2011) and, from an axiomatic point of view, (Heyting 1980).

3 The prehistory of projective geometry is discussed in Kline (1972, p. 285 ff.) and, with a focus on its roots in the renaissance, in Andersen (2007).

4 Poncelet’s justification crucially relied on his notorious *principle of continuity*, which is discussed, for example, in Nagel (1939, p. 151 ff.) and Gray (2007, p. 43 ff.).

5 For example, both Jakob Steiner and later Von Staudt gave purely projective definitions of the conic sections that make no use of metrical notions or coordinates. See Coxeter (1987, pp. 71–80) for a systematic and Coolidge (1934) for a historical discussion.

6 See Plücker (1831), Möbius (1827). The idea is to represent projective points of the plane by triples of numbers with the stipulation that two triples represent the same point if one results from the other by scalar multiplication. In a similar way, one can introduce *line coordinates*, which are also triples of numbers, again, with the understanding that two triples represent the same line if one results from the other by scalar multiplication.

7 See Klein (1926, p. 115 ff.) for discussion.

8 See Klein (1872, 1873) and Gray (2005), A’Campo and Papadopoulos (2015) for discussion.

9 This way of interpreting homogeneous coordinates has become standard and is adopted in many modern textbooks. Although this way of looking at things seems obvious, the first textbook-style presentation I was able to find is in Klein (1908, pp. 92 ff.).

10 See Pasch (1882), Peano (1894), Fano (1892), Pieri (1894–95), Veblen and Young (1908) and Veblen and Young (1910). See Torretti (1978, p. 188 ff.), for further discussion. For a closer discussion of the development of projective geometry in Italy, see Avellone et al. (2002).

11 The relevant writings of this period are reprinted in Frege (1984). Frege’s second major area of specialization was complex analysis. Although he did not publish anything on this subject, Frege lectured extensively on several issues in complex analysis, such as Abelian integrals and the Riemannian approach to complex analysis. For a list of his courses, see Kreiser (2001, p. 280). In this article I will not discuss the complicated issue of complex analysis in nineteenth century and how Frege might be positioned in nineteenth century debates about the foundations of this subject. See Tappenden (2006) and Shipley (2015) for more on this.

12 See, for example, Blanchette (1996, 2007, 2012), Ricketts (1997), Resnik (1974), Tappenden (2000, 2005).

13 Detailed studies of Desargues’ theorem, as well as its historical and philosophical significance, can be found in Dawson (2015) and in Arana and Mancosu (2012).

14 See, e.g., Von Staudt (1847, p. 24), Reye (1866, p. 16), Thomae (1873, p. 2), and Mancosu (2015, 2016) for further discussion.

15 Von Staudt also seems to have coined the term “orientation” (“Stellung”) for what is common to parallel planes [an alternative translation of “Stellung” is “aspect”, see Mancosu (2016, p. 60ff.)], an expression that is also mentioned by Frege in his *Grundlagen*. That Frege might have written the introduction to his dissertation with Von Staudt in mind gains additional support from some of his suggestions in connection with imaginary elements, which we will discuss in the upcoming sections. Mark Wilson has already pointed out some of the similarities between Frege and Von Staudt in his (Wilson 1992, 2010).

16 As far as I am aware, Frege’s dissertation has not been discussed in detail in the secondary literature, although some of it is discussed in the very informative (Shipley 2015).

17 Frege defines the ‘distance’ between two imaginary points $P_1(x_1, y_1)$ and $P_2(x_2, y_2)$ by extending the formula for Euclidean distance

$$d(P_1, P_2) := \sqrt{(x_2 - x_1)^2 + (y_2 - y_2)^2}$$

to complex numbers and explicitly notes that “what is essential to the concept of distance is not the intuitive character of a straight line but conformity to the laws of algebraic analysis” (Frege 1984, pp. 9–10).

18 According to Von Staudt, the central problem with the analytic approach is that by defining imaginary points in terms of coordinates we merely “transfer the language of algebra to geometry”. But “[w]here is”, von Staudt then asks, “the imaginary point if one abstracts from the coordinate system?” See Von Staudt (1856, IV). Russell, who thought that imaginary points are “destitute of philosophical meaning” (Russell 1897, p. 43), described the same issue in his characteristic wit as follows: “As well might a postman assume that, because every house in a street is uniquely determined by its number, therefore there must be a house for every imaginable number” (Russell 1897, p. 44). Von Staudt’s construction is explained in more detail, e.g., in Klein (1908, p. 133 ff.) and its broader significance, specifically in connection with Frege, is discussed by Wilson in his (Wilson 1992, 2005, 2010).

19 The distinction between *definition* and *representation* which is made here is not necessarily a sharp one. See, for example, Klein’s discussion of Von Staudt in Klein (1926, p. 136), where Klein, in our current terminology, reads Von Staudt as providing merely a *geometrical representation* (“geometrisches Abbild”) rather than a *reductive definition*.

20 Frege’s proposal in the *Grundlagen* (which we will discuss in more detail in section 9) is to introduce points at infinity as classes of parallel lines. In his correspondence with Frege, Pasch suggests that Frege’s definition may well be insufficient since “imaginary points are still to come” (Frege 1980, p. 106), indicating that he thinks that Frege’s method might not be generalizable to account for imaginary elements. Unfortunately, Frege’s reply to Pasch is lost, and Pasch’s subsequent reply to Frege does not help us in reconstructing any definite views. See again Wilson (1992, 2005) for further discussion of the Von Staudt approach in connection with Frege.

21 See Frege (1984, p. 3). The translation of this passage follows the lead of Jamie Tappenden, who correctly notes that the translation of “bloße Übertragung” by “carrying over” obscures the connection to transfer principles, such as Hesse’s “Übertragungsprinzip”. See Tappenden (2005, p. 222).

22 Note that, from a modern perspective, making a distinction between ‘ordinary points’ and ‘points at infinity’ is already a departure from a purely projective point of view, because the property of ‘being a point at infinity’ is not preserved under projection. As Veblen and Young note, “[i]n projective geometry, then, there can be no distinction between the ordinary and the ideal elements of space” (Veblen and Young 1910, p. 14). Frege certainly *seems* to be expressing such a point of view in the quoted passage. However, we will see later on that he did not consistently stick to this view after all.

23 That Frege’s example is problematic is also mentioned in Schirn (2019, p. 933 ff.), the only article I am aware of in which the example is discussed in some detail. Although Schirn’s discussion of the ‘punctured sphere’ is not entirely clear to me, his conclusion that the structure induced by Frege’s projection is not homeomorphic to the real projective plane is certainly correct. As we saw, the projection mapping is not even one-to-one.

24 The concept of ‘transfer’ is also mentioned by Frege in his discussion of the relationship between real and imaginary forms, where he makes clear, however, that we can transfer truths from the real plane to the imaginary plane in virtue of the algebraic properties that are shared by the real and the imaginary numbers (Frege 1984, p. 2).

25 Expressions like “bundle of lines” or “pencil of lines” (“Geradenbündel”, or “Geradenbüschel”) belonged to the standard vocabulary of nineteenth century geometers in Germany. See again Mancosu (2015).

26 Both the real projective plane and space have a lot of weird properties, which is why Torretti calls projective space a “thoroughly unintuitive entity” (Torretti 1978, p. 110). Partial visualizations of the real projective plane are discussed in Apéry (1987). For a discussion of the beginnings of topology, see chapter 50 of Kline (1972), especially Sect. 3.

27 See Nagel (1939), Gray (2007, p. 53 ff.) and Lorenat (2015) for a discussion of the duality controversy. Some of the issues that are discussed in this section have been investigated in Eder (2019), which in turn is partly based on joint work with Georg Schiemer [see Eder and Schiemer (2018)].

28 Chasles later clarified that really all that matters is that incidence is preserved. So it is not required that applying the transformation twice leads back to the original configuration, as in Poncelet’s version that is based on the theory of poles and polars. See Chasles (1837, §§34–35).

29 See Gray (2007, p. 53 ff.) for further discussion.

30 A central reason for Pasch’s superior understanding consists in his explicit axiomatic point of view, where we start with a small number of “stem concepts” and “stem propositions” for projective geometry; further concepts are then introduced definitionally and theorems are proved purely deductively from the stem propositions plus definitions. For further discussion of Pasch’s mathematics, see Torretti (1978, p. 210 ff.). Pasch’s unique philosophy of geometry, including his empiricism, is further discussed in Schlimm (2010).

31 Transfer principles were discussed from a broader, methodological point of view in the context of geometry, for example, by Hermann Hesse in his (Hesse 1866, p. 32) and subsequently played a prominent role in Klein’s famous ‘Erlangen Program’ (Klein 1872). See Schiemer (2020) for further discussion of transfer principles in connection with Klein. The significance of transfer principles has been emphasized in the context of a discussion of Frege’s views on ‘metatheory’ by Jamie Tappenden in his Tappenden (2005, p. 193).

32 See, for example, Frege’s “Lecture on the Geometry of Pairs of Points in the Plane” (Frege 1984, p. 103 ff.), which is also discussed in Shipley (2015), Tappenden (1995, p. 326), and Wilson (2010, p. 402).

33 See Klein (1872, p. 70) and Klein (1873), especially Klein (1873, pp. 120–121). For further discussion, see Gray (2005) and A’Campo and Papadopoulos (2015).

34 Similarly, in his “Review of Hoppe, Lehrbuch der analytischen Geometrie I” from 1880, Frege notes that the projective properties are those that are “preserved under a collinear transformation” (Frege 1984, p. 102).

35 Note that, while unproblematic for most *mathematicians*, the Cayley-Klein reduction of metrical Euclidean geometry to projective geometry was not uncontroversial for some *philosophers*. Russell famously claimed that this reduction is “purely technical” and of no philosophical significance, due in part to the fact that it relies on the presence of imaginary points (Russell 1897, p. 41 ff.). As we saw in our discussion of Frege’s dissertation, these specific worries were not an issue for Frege though. Frege had no quarrels with imaginary forms in geometry.

36 This is the problem that identity statements of the form “the direction of *a* = *c*” are not settled by the stated identity criterion for directions (parallelism), which can only be applied when both sides of the identity statement are given in the form “the direction of *a*”. See Heck (1997) for further discussion of this problem.

37 See Frege (1980, p. 106), where Pasch responds to a proposal apparently made by Frege in an earlier letter. As Mancosu has shown in detail in Mancosu (2015, 2016), similar proposals had been made by other nineteenth century geometers, although Frege was arguably more explicit than earlier geometers with his identification of directions with classes of parallel lines. In his (Mancosu 2016), Mancosu also develops a systematic account of abstraction and its role in mathematics.

38 In his (Wilson 1992), which influenced much of the recent work on Frege’s mathematical background in geometry, Mark Wilson has highlighted an important distinction between two views on extension elements. According to the first view, the main purpose of extension elements is to enable us to state facts about ordinary Euclidean objects, the objects we “really care about” (Benacerraf), in a more convenient way. By contrast, according to the second view, these objects are considered to be part of the ‘ultimate reality’ in which ordinary geometrical objects live. Now, I have my doubts about Wilson’s assessment that geometers “saw the additions as revealing the ‘true world’ in which geometrical figures live” (Wilson 1992, p. 153) if this is supposed to be a judgement about *all*, or even *most*, nineteenth century geometers. In any case, I agree that a “recasting of geometrical ontology” took place in Frege’s *Grundlagen*. But while it is true that Frege’s definitions were in part intended to legitimize antecedent mathematical practices, I think it is misleading to characterize this as an indication of some “hidden essentialism”, if that is supposed to suggest a commitment to a ‘true world’ in which geometrical figures live *beyond* Euclidean space. Frege is quite forthright about his view that there is only *one* space in which geometrical figures live, namely, *Euclidean space*.

39 Interestingly, Frege also hints at the possibility of introducing objects that arise by abstracting from geometrical configurations that are related to each other by a collineation (Frege 1884, p. 75). The abstract entities that are introduced in this way are something like ‘abstract structures’ which are instantiated by various projectively isomorphic configurations.

40 The procedure described here is well-known nowadays and contains a general template for constructing a projective plane from a given affine plane. See Beutelspacher and Rosenbaum (1998, p. 27 ff.).

41 Note that the obvious proofs of these propositions make use of the axiom of parallels. Presumably, Frege would not be bothered by such uses of the axiom of parallels. However, since projective geometry was considered to be the common basis for *all* of geometry, many of Frege’s contemporaries would have disagreed and required that projective geometry be neutral with respect to the status of the axiom of parallels. See, for example, Pasch’s discussion in the introduction to his *Vorlesungen*, where he credits Felix Klein with the insight that projective geometry is “independent of the theory of parallels” (Pasch 1882, IV).

42 In the case of the arithmetic of natural numbers, Frege’s logicism required him to show that something like the Dedekind-Peano axioms can be proven from pure logic plus definitions. Similarly, Frege’s relative logicism with respect to projective geometry would require him to show that each proposition in some system of basic principles for projective geometry can be proven from the axioms of Euclidean geometry plus definitions. Hence, this strategy presupposes a precise delineation of the basic principles of projective geometry. As we saw earlier, these kinds of investigation were pursued with rigour only towards the end of the nineteenth century, starting with Pasch (1882). They were not pursued by Frege.

43 See, in particular, the manuscript *Logik in der Mathematik* from 1914 (Frege 1979, p. 203).

44 Frege’s articles are reprinted in Frege (1984).

45 Frege presents his approach in Frege (1984, pp. 333–339). See Tappenden (2000, 2005), Ricketts (1997) and Blanchette (2012, ch. 5) for further discussion.

46 It is important to keep an eye on the differences between various conceptions of duality. In particular, as I have argued elsewhere, if Tappenden is right that Frege’s 1906 proposal on independence proofs is based on his understanding of duality, then, contrary to his own intentions, this provides evidence *against* his general narrative that informal model-theoretic reasoning was not foreign to Frege. See Tappenden (2005) and section 4.3 of Eder (2019) for further discussion of this point.

47 Remember that the often-quoted passage from Pasch’s *Vorlesungen*, where he states that “if geometry is to be genuinely deductive, then the process of inferring must be everywhere independent of the meaning of the geometrical concepts” (Pasch 1882, p. 98) is part of Pasch’s discussion of duality in §12. Pasch had mentioned the *Vorlesungen* in the course of his correspondence with Frege from around 1904. So it is highly unlikely that Frege did not look into this book at that time.

48 Frege took courses on philosophy with Kuno Fischer in Jena and with Hermann Lotze in Göttingen. The influence of Lotze and other German philosophers of the time is investigated in more detail in Sluga (1980) and Gabriel (2002).

49 Hans Sluga and Michael Dummett are the most prominent ones. See Sluga (1980), Dummett (1991), and, more recently, Schirn (2019).

50 Kant characterized analytic judgements in different ways that are not equivalent from Frege’s point of view. According to one characterization, analytic judgements are ones where the predicate concept is contained in the subject concept. Given Frege’s new analysis of judgements, this criterion is not always applicable, because not all judgements have a subject-predicate form. As Frege makes clear, his own characterization is supposed to capture more precisely what Kant had in mind when he was calling analytic judgements “judgements of clarification” (“Erläuterungsurteile”). Roughly, the common ground is that both thought that analytic judgements are ones that can be reduced to logical truths by means of conceptual analysis. See Frege (1884, 3 ff. and 17 ff.) and Kant (1781/1787, p. 130).

51 See Kant’s discussion in (Kant, 1781/1787, p. 630 ff.). That Kant assigned an indispensable role to intuition in *proofs* is by some interpreters traced to the fact that Kant had no precise axiom systems to work with. Thus, intuition is required to fill the gaps. See Friedman (1985) and Kvasz (2011) for further discussion of Kant’s philosophy of geometry.

52 According to Frege, on the first conception the role of intuitions as *individual* representations (as opposed to *general* representations, i.e., *concepts*) is emphasized. On the second conception, intuitions are more closely tied to the faculty of *sensibility*. This contrast is also reflected in modern discussions about the nature of Kantian intuition(s). See especially the debate between Hintikka and Parsons [reprinted in the collection (Posy 1992)] and (Friedman 1985).

53 In connection with Frege, this point has been made in Dummett (1991), although the same point was discussed already in Frege’s time. See, e.g., Torretti (1978, p. 149 ff.). This is probably why Schirn trusts that “Frege was aware of this but presumably thought that he could ignore this in his account” (Schirn 2019, p. 956).

54 In *Über Sinn und Bedeutung* Frege makes the same argument with respect to *ideas* (“Vorstellungen”), noting that “an exact comparison is not possible, because we cannot have both ideas together in the same conciousness” (Frege 1984, p. 160).

55 According to Kant, “[s]pace is not something objective and real, neither substance, nor accident, nor relation; but subjective and ideal, arising by fixed law from the nature of the mind” (Kant 1894, p. 65, 66). As such, however, space is also ‘objective’ in the sense of being a feature that is common to *all* Human beings. Russell once summed up a common understanding of Kant by saying that “to Kant, who was nothing of a psychologist, a priori and subjective were almost interchangable terms” (Russell 1897, p. 2).

56 In his *Logic in Mathematics* from 1914, apparently still under the impression of his controversy with Hilbert, Frege seems to endorse something of that sort. He says: “When a straight line intersects one of two parallel lines, does it always intersect the other? This question, strictly speaking, is one that each person can only answer for himself. I can only say: so long as I understand the words ‘straight line’, ‘parallel’ and ‘intersect’ as I do, I cannot but accept the parallels axiom. If someone else does not accept it, I can only assume that he understands these words differently.” (Frege 1979, p. 247).

57 Some interpreters have tried to find a way though. See, e.g., Dummett (1991) and, more recently, (Shipley 2015, p. 2). In his (Schirn 2019), Schirn also provides a discussion of several issues related to §26, and I fully agree with him that it is not clear “how the claimed dependence of the validity of the (objective) geometrical axioms on the nature of our faculty of intuition [$\ldots$] could be reconciled with the subjectivity of our spatial intuitions” (Schirn 2019, p. 959).

58 Reye, for example, notes that in Von Staudt’s approach geometrical knowledge is acquired by “immediate intuition” (Reye 1866, VIII). For more on “projective intuition”, especially in Klein’s work, see Torretti (1978, p. 147).

59 Immediately before this passage, Frege mentions spaces of four dimensions or positive curvature, referring to contemporary investigations of non-Euclidean geometry. Models of non-Euclidean geometry had been devised in Beltrami (1868) and Klein (1871). For further discussion of Frege’s conception of intuition in the context of models of non-Euclidean geometry see Schirn (2019, p. 941).

60 See Torretti (1978, p. 188 ff.) for more on the development of modern axiomatic geometry, and Torretti (1978, p. 137 ff.) and Gray (2005) for further discussion of Klein’s approach.

61 See Wilson (2010) and Shipley (2015) for further discussion of Frege’s views on ‘recarving’ space.

62 Frege explains his views in detail in the second part of his series of papers on the foundations of geometry from 1906 Frege (1984, p. 293 ff.). See also Frege (1980, p. 182 ff.) and Frege (1980, p. 57 ff.), where Frege discusses ‘algebraic axiom systems’. For further discussion see Blanchette (1996, 2007, 2012), Resnik (1974), Ricketts (1997), and Tappenden (2000).

63 In an unpublished fragment titled “On Euclidean geometry”, Frege writes that “[w]hoever acknowledges Euclidean geometry to be true must reject non-Euclidean geometry as false, and whoever acknowledges non-Euclidean geometry to be true must reject Euclidean geometry” (Frege 1979, p. 169), and he rhetorically asks: “Do we dare to treat Euclid’s Elements, which have exercised unquestioned sway for 2000 years, as we have treated astrology?” An enlightening discussion of Frege’s views on axioms and independence proofs in geometry, and how they evolved over time, can be found in Tappenden (2000).
